# Temperature and pH Stimuli-Responsive System Delivers Location-Specific Antimicrobial Activity with Natural Products

**DOI:** 10.1021/acsabm.3c00588

**Published:** 2023-12-11

**Authors:** Gareth Morris, Sean Goodman, Ioritz Sorzabal Bellido, Chiara Milanese, Alessandro Girella, Piersandro Pallavicini, Angelo Taglietti, Mattia Gaboardi, Frank Jäckel, Yuri A. Diaz Fernandez, Rasmita Raval

**Affiliations:** † Open Innovation Hub for Antimicrobial Surfaces, Surface Science Research Centre, University of Liverpool, Liverpool L69 3BX, U.K.; ‡ Department of Chemistry, University of Pavia, Via Taramelli 12, Pavia 27100, Italy; § Materials Physics Center, CSIC-UPV/EHU, Donostia - San Sebastian 20018, Spain; ∥ Department of Physics and Stephenson Institute for Renewable Energy, University of Liverpool, Liverpool L69 7ZE, U.K.

**Keywords:** stimuli-responsive materials, controlled release, fatty acids, antimicrobial surfaces, medical devices

## Abstract

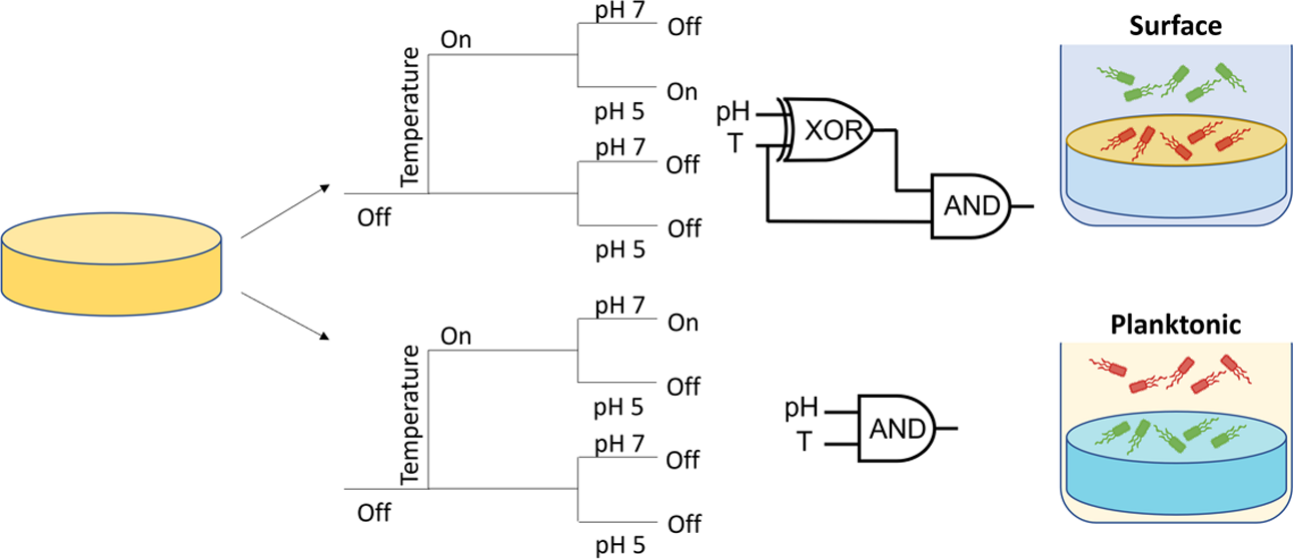

Smart materials with controlled stimuli-responsive functions are at the forefront of technological development. In this work, we present a generic strategy that combines simple components, physicochemical responses, and easy fabrication methods to achieve a dual stimuli-responsive system capable of location-specific antimicrobial cargo delivery. The encapsulated system is fabricated by combining a biocompatible inert polymeric matrix of poly(dimethylsiloxane) (PDMS) and a bioactive cargo of saturated fatty acids. We demonstrate the effectiveness of our approach to deliver antimicrobial activity for the model bacteria *Escherichia coli*. The system responds to two control variables, temperature and pH, delivering two levels of antimicrobial response under distinct combinations of stimuli: one response toward the planktonic media and another response directly at the surface for sessile bacteria. Spatially resolved Raman spectroscopy alongside thermal and structural material analysis reveals that the system not only exhibits ON/OFF states but can also control relocation and targeting of the active cargo toward either the surface or the liquid media, leading to different ON/OFF states for the planktonic and sessile bacteria. The approach proposed herein is technologically simple and scalable, facing low regulatory barriers within the food and healthcare sectors by using approved components and relying on fundamental chemical processes. Our results also provide a proof-of-concept platform for the design and easy fabrication of delivery systems capable of operating as Boolean logic gates, delivering different responses under different environmental conditions.

## Introduction

The use of natural products as antimicrobial agents has emerged as an important strategy to address the rapid threats of antimicrobial resistance and viral pandemics that are increasing faster than the ability of pharmaceutical companies to produce new drugs.^1−8^ Among naturally occurring bioactive molecules, fatty acids represent a family of compounds that are present in the human skin^9−11^ and in many natural products,^10,12,13^ suitable for human and animal consumption,^14,15^ and widely approved as food additives and cosmetic ingredients within commercial formulations.^15−20^ Therefore, they have low regulatory barriers for medical and pharmaceutical applications. Specifically, fatty acids display antimicrobial properties^9,10,13,21−26^ and have been used since antiquity as food preservatives^14,27^ and antiseptics.^12,28^

Delivering such natural product antimicrobial compounds via a stimuli-responsive system would provide a promising route toward smarter antimicrobial technology applications. Encapsulation approaches based on phase-transition processes are versatile and provide reliable routes for delivering active functions in a controlled fashion. Generally, the material experiencing the phase transition is used as the encapsulating unit able to release an active cargo at specific transition temperatures.^29−31^ Similarly, other control release systems have been demonstrated using pH-sensitive encapsulation matrixes,^29,32−35^ biodegradable liposomes,^34,36−40^ and mesoporous hard materials.^41−46^

Herein, we exploit a different strategy by using an inert and biocompatible encapsulation matrix based on poly(dimethylsiloxane) (PDMS) with the ability to load and release the active component (fatty acid) under controlled conditions. In this case, the thermal responsivity of the system is delivered by the phase transition of the active component itself, while the encapsulation matrix acts as a carrier and mechanical barrier to prevent the passive release of the cargo into the media. We combine spatially resolved spectroscopy and thermal and structural material analysis to probe the mode of action of the system. Our work shows that this system is able to deliver stimuli-responsive antimicrobial activity for the model bacteria *Escherichia coli* both directly at the surface and on planktonic bacteria and operates in a manner that resembles Boolean AND and AND/XOR logic gates.

## Results and Discussion

### Design of the Responsive Systems Based on PDMS Encapsulation

Temperature and pH are suitable triggering signals for many processes in medical and industrial applications. Here, we demonstrate an easy to implement approach for the controlled release of bioactive components from PDMS, triggered by temperature and pH cues. The choice of PDMS as the encapsulation matrix was driven by the strong ability of this material to undergo swelling in the presence of some organic liquids and its intrinsic low permeability to water.^47−50^ PDMS is also widely used in technology (e.g., medical devices^51−60^) and in the food industry (regulated food additive E900)^17^ and is therefore amenable to translation into technologically relevant applications.

**Figure 1:**
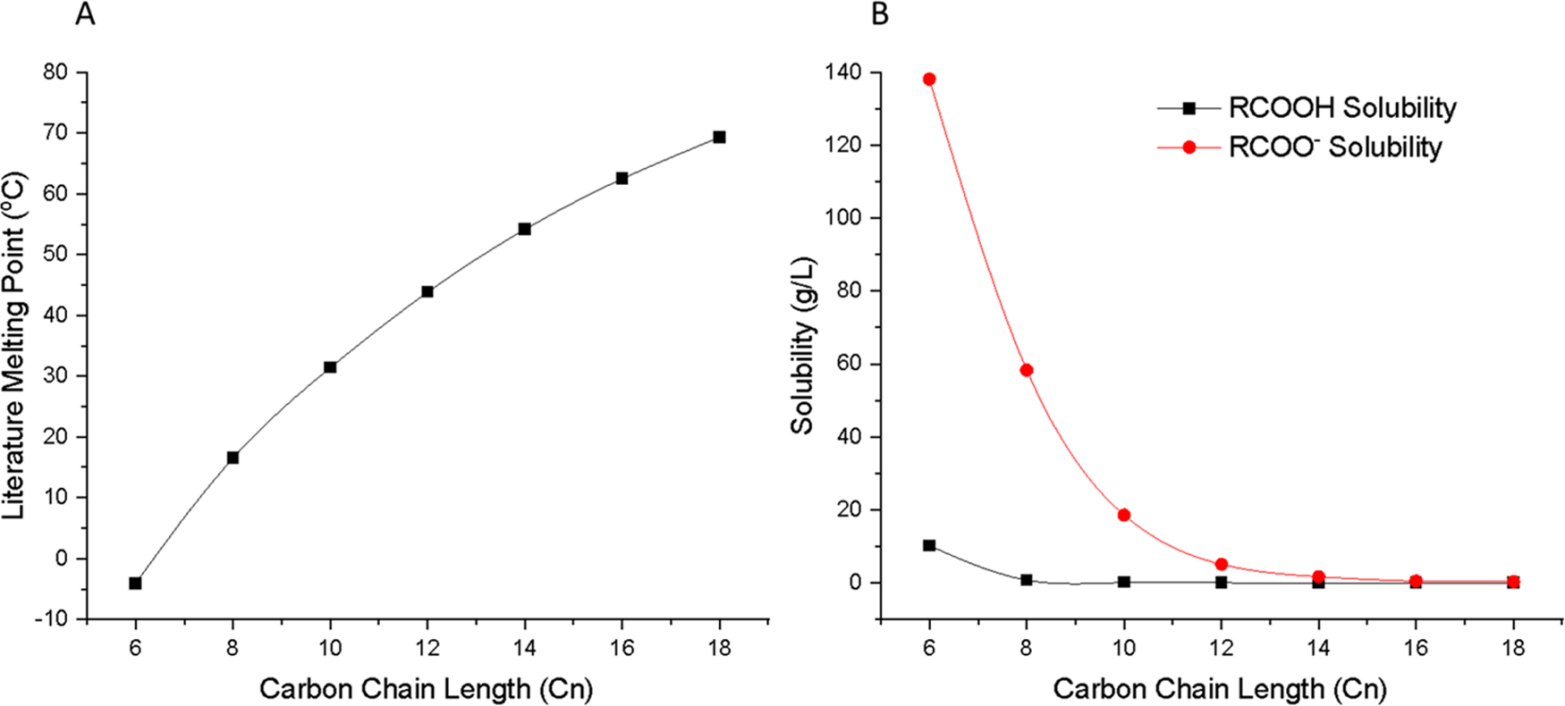
Melting point (A) and water solubility (B) of fatty acids as functions of the carbon chain length. Cn is the total number of carbons. R refers to the linear aliphatic chain.^61−64^

The active cargo components chosen for this work were saturated fatty acids. This family of molecules also has interesting physical and chemical properties. The melting points of different fatty acids increase monotonously as the carbon chain length of the molecule increases (Figure 1a), providing a direct route to control the temperature of the triggered response by simply choosing the molecule of appropriate carbon chain length. Additionally, the solubility of long-chain aliphatic acids in water is pH-dependent (see Figures 1b and SI1): at acidic pH, the molecules are mainly present in the protonated carboxylic acid state, displaying low solubility in water; while at basic pH, well above the p*K*_a_ of the carboxylate, the solubility increases by at least 2 orders of magnitude.^61−64^ In this work, we exploit these two inherent properties to create a smart antimicrobial system able to respond simultaneously to signals of temperature and pH.

In the following sections, we will combine complementary analytical techniques to show how PDMS materials can be effectively loaded with sufficient amounts of fatty acids that can be subsequently released under specific pH and temperature conditions, delivering a targeted antimicrobial response.

### Fabrication of the Responsive Systems

Loading of fatty acids into the encapsulation system was achieved by exposing PDMS samples to the fatty acids at temperatures above the specific melting point of each compound (Table 1). Under these conditions, the liquid fatty acids act as solvents, inducing a swelling process within the PDMS and penetrating the polymeric matrix. When the system is cooled abruptly to below the melting point of the fatty acid, the fatty acid is trapped in its solid state inside the PDMS matrix. Any surface excess is removed by quick rinse with a poor-swelling solvent (e.g., ethanol),^47^ thereby leaving a self-sealed out-of-equilibrium encapsulated system. The PDMS samples can, in principle, be produced with both macroscopic and microscopic dimensions and in any desirable shape, exploiting molding and soft-cutting techniques suitable for industrial scale-up. The typical samples used for this work were disks with a thickness of 1 mm and a diameter of 40 mm (or 7 mm diameter for bacteriological experiments).

**Table 1:** Loading of Fatty Acids into PDMS^a^

fatty acid	melting point (°C)	loading temperature (°C)	mg fatty acid loaded per g of PDMS	% weight of fatty acid loaded into PDMS
decanoic (C_10_)	32.7 (±0.1)	37.0 (±0.1)	89 (±12)	8.9 (±1.2%)
lauric (C_12)_	45.2 (±0.3)	50.0 (±0.1)	58 (±9)	5.8 (±0.9%)
myristic (C_14_)	55.4 (±0.6)	60.0 (±0.1)	37 (±7)	3.7 (±0.7%)

a Errors are reported between brackets.

Following this strategy, encapsulation of three different saturated fatty acids was achieved, namely, decanoic (C_10_), lauric (C_12_), and myristic (C_14_) acids, with distinct melting temperatures in a range between 30 and 60 °C, according to differential scanning calorimetry (DSC). The amount of each fatty acid loaded into the PDMS encapsulation matrix was determined by gravimetric analysis, obtaining between 9 and 3% increase in weight for the different fatty acids (Table 1).

### Characterization of the Encapsulated Systems

**Figure 2:**
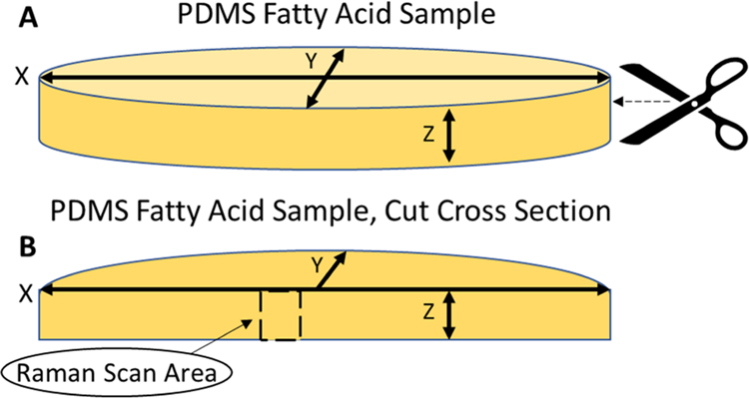
Schematic showing an area of the sample cross section mapped using Raman. (A) Schematic of a full sample showing where the samples were cut prior to Raman cross-sectional experiments. (B) Schematic showing an example of a single cross-sectional area used for Raman data collection. For each sample, cross sections were taken over multiple regions of interest across the cut interface.

**Figure 3:**
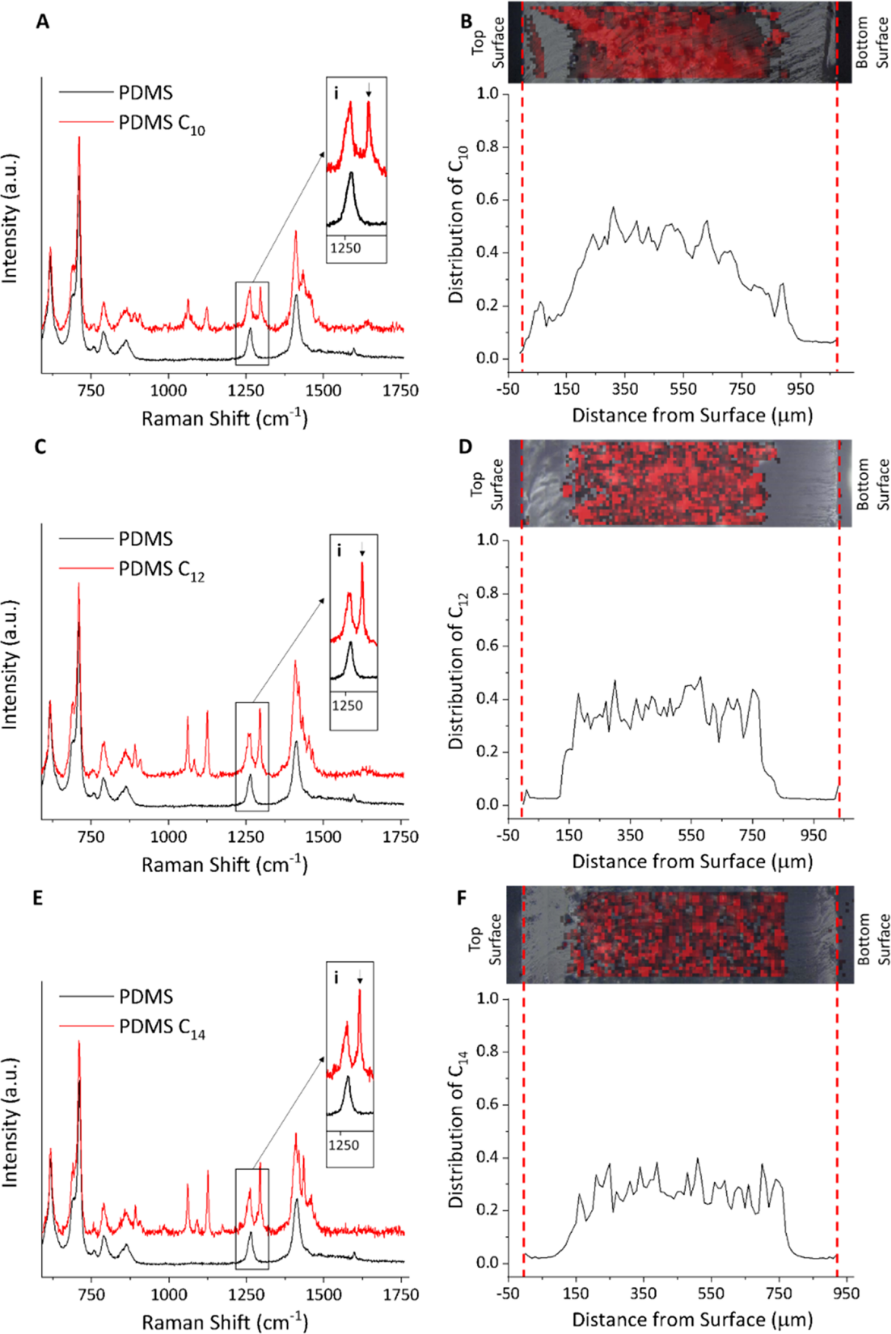
Representative cross-sectional Raman data for PDMS and PDMS loaded with fatty acids. (A, C, and E) Raman spectra for decanoic, lauric, and myristic acids, respectively; the insets highlight the distinct τCH_2_ Raman band chosen for mapping the distribution of the cargo. (B, D, and F) Cargo distribution profiles across the cross section of the loaded samples, demonstrating the successful encapsulation of the cargoes within the polymeric matrix of PDMS.

Raman spectroscopy was used to assess the depth distribution of the fatty acids in cross sections of the encapsulated samples across the *z*-direction (Figure 2). Successful encapsulation of the fatty acids was proved by spatially resolved Raman spectroscopy since the individual system components display characteristic Raman spectra (Figures 3, SI5, and SI6). Specifically, the Raman spectra of the fatty acids show a distinct peak at 1295 cm^–1^ typical of the τCH_2_ twist vibration^65^ of the alkyl chain (Figures 3, SI2, 5, and 6). This band appears in a spectral region that is clear from Raman peaks arising from the PDMS encapsulation matrix, thus enabling the fatty acid distributions across the samples to be mapped with a Raman confocal microscope.

Our data demonstrate that the fatty acid cargo of the fabricated systems is successfully encapsulated and located predominantly within the matrix, in a confined region that is ∼50–150 μm away from the sample surface (Figure 3b,d,f). The low concentration of fatty acid near the surface region of the encapsulated systems can be attributed to the efficient rinse-off of the surface excess by quick immersion in cool ethanol. Even though ethanol is a poor-swelling solvent for PDMS,^47^ the high solubility of fatty acids in this solvent^61^ allows for a fast, local solubilization at the interface, efficiently removing the excess of cargo from the near-surface region of the material.

Additionally, the lateral XY distribution of fatty acids within the samples was evaluated by collecting over 100 Raman spectra across a regular grid with data points collected at a depth of 500 μm under the surface (Figure SI10a). For encapsulated decanoic, lauric, and myristic acids, the Raman data show a fairly even spread of the cargo at this depth leading to narrow variations in the distributions, with 90% of the data points within two standard deviations from the mean intensity values (Figure SI10b–d). Overall, the narrow statistical distributions of Raman intensities for the three fatty acids suggest a largely homogeneous spatial distribution of the active cargoes within the bulk for all of the encapsulated samples, surrounded by a cargo-free zone maintained 50–150 μm from the surface.

### Wettability and pH Response of the Loaded Samples

**Figure 4:**
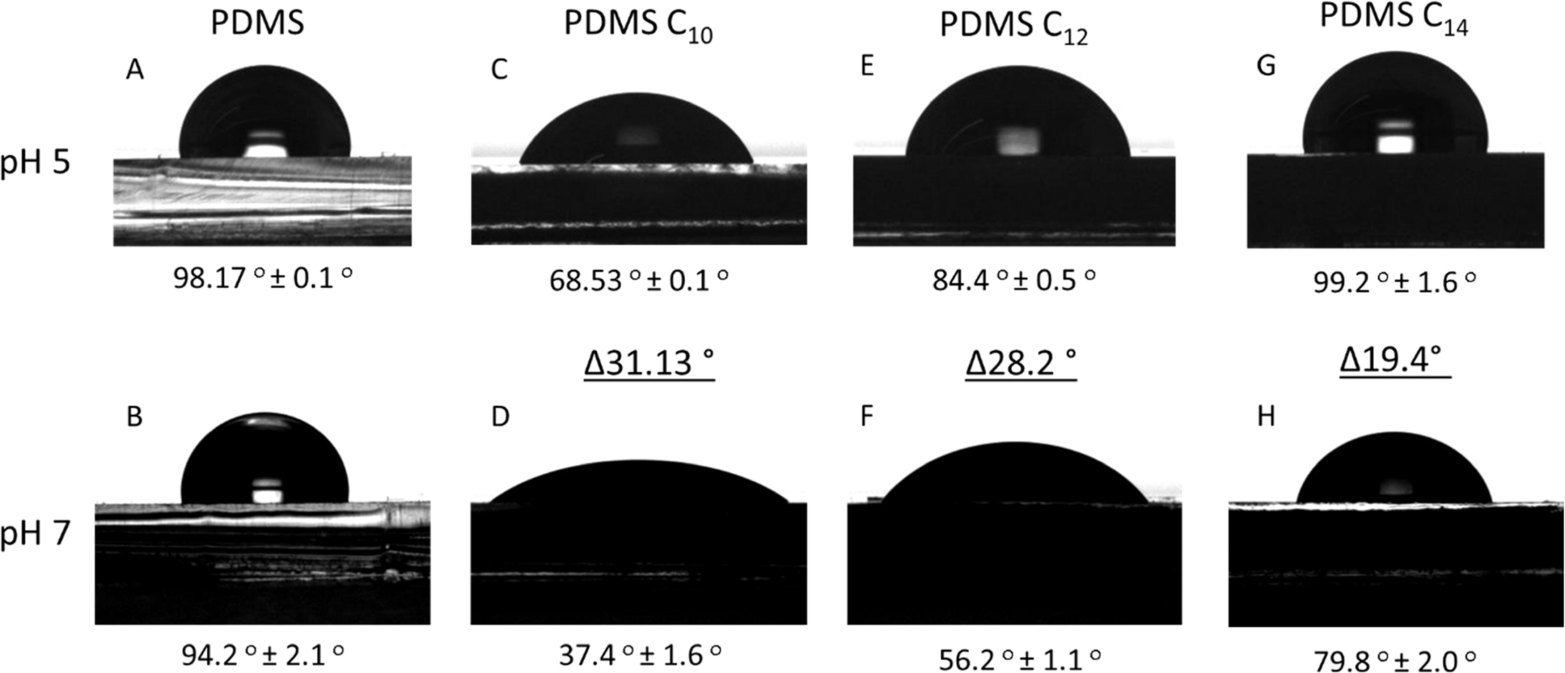
Contact angle at different pH values for pristine PDMS (A, B), PDMS C_10_ (C, D), PDMS C_12_ (E, F), and PDMS C_14_ (G, H). Delta values show the difference in contact angle upon change of pH.

The wettability of the encapsulated systems was investigated by determining the static contact angle of water at acidic and basic pH values. Pristine PDMS is well-known for being inert and highly hydrophobic, as confirmed by the high contact angles observed for both acidic and basic pH media (Figure 4a,b). However, the contact angle of the encapsulated systems displayed a pH-responsive behavior, showing hydrophobic properties at acidic pH, which turns into a more hydrophilic response at pH = 7 (Figure 4c–f). This wettability behavior can be explained based on the surfactant properties of long-chain fatty acids that are further enhanced at basic pH conditions due to the deprotonation of the carboxylic group. The reduction of the liquid–solid and liquid–air interfacial tensions due to the presence of the deprotonated fatty acid molecules at pH = 7 is responsible for the enhanced wettability observed at physiological pH. It is interesting to note that our Raman cross-sectional data showed no discernible signals of fatty acids at the surface of the dry samples (Figure 3), however, it is possible that a small residual amount of fatty acids is still present at the surface but remains below the detection limit of the Raman microscope. However, we also note that it is not straightforward to correlate the Raman data of the saturated samples, which are collected in dry conditions, with the wettability data that show the response of a drop of water on the unloaded and loaded samples. For example, simulation of the dissolution of organic molecules in a thin cross-linked PDMS membrane model shows that the organophilicity of PDMS causes it to swell in organic solvents,^68^ as would be the case with the fatty acids used in our work. This alters the structure of the PDMS and affects the void size distribution (VSD) and the structure at the surfaces, which would directly affect the wettability.

The pH-responsive hydrophobicity switch emerges as a useful property for our encapsulation systems. First, the low wettability of the surface of PDMS at acidic pH ensures reduced solvent exposure for the cargo and, consequently, lower release. Conversely, the increased wettability at physiological pH ≈ 7 will have the opposite effect, facilitating the release of the cargo. All three fatty acids displayed this pH-dependent contact angle behavior, although the absolute values depended on the nature of the encapsulated fatty acid. Our data suggest that at acidic pH, the encapsulated systems remain largely isolated from the aqueous media due to the high interfacial tension between water and PDMS. This situation is reversed at physiological pH ≈ 7, where water wettability increased considerably.

### Thermal Response/Behavior of the Encapsulated Systems

The thermal behavior of the fatty acids within the encapsulation matrix was investigated using differential scanning calorimetry (DSC), grazing-incidence synchrotron X-ray powder diffraction (GIXRD), and Raman spectroscopy to obtain information on the melting transitions, crystallinity, and spatial distribution in the encapsulated state.

DSC data shows that pure PDMS is thermally inert in the temperature range from −70 to + 80 °C, showing no signals associated with thermal transitions (Figure SI11). However, pure fatty acids display melting-point transitions at distinct temperatures related to the length of the aliphatic chain (Tables 2, SI2 and Figures S12–SI14). These transitions are also observed for the fatty acids encapsulated within PDMS, but the onset temperatures for melting are systematically lower with respect to the pure fatty acids and the calorimetric signals are also broader (Tables 2, SI2 and Figures S12–SI14).

**Table 2:** Differential Scanning Calorimetry (DSC) Onset of Melting Data for Pure and PDMS-Encapsulated Decanoic, Lauric, and Myristic Acids

	sample type	average *T*_melt_ (°C)
decanoic (C10)	pure	32.7 (±0.1)
	encapsulated	29.8 (±0.4)
lauric (C12)	pure	45.2 (±0.3)
	encapsulated	37.6 (±1.7)
myristic (C14)	pure	55.4 (±0.7)
	encapsulated	45.7 (±3.6)

The difference between pure and encapsulated fatty acids increases with the length of the fatty acid chain. An inherent level of inhomogeneity is also present in the encapsulated fatty acid system, leading to broader DSC peaks with locally different melting points appearing at different heating times within the DSC profiles. These results suggest the presence of a weak interaction between the fatty acid and the polymeric matrix, like in a solvent–solute system, decreasing the average melting temperature of the solutes (freezing point depression). Overall, these results demonstrate that the encapsulated systems should be capable of delivering a thermal response arising from the melting transition of the fatty acid cargo.

**Figure 5:**
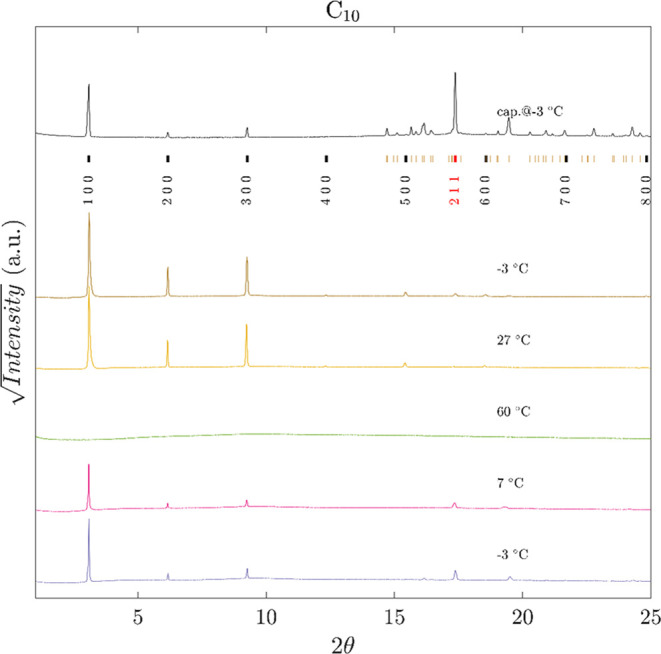
*In situ* GIXRD patterns of C_10_-encapsulated samples recorded at different temperatures upon heating (−3, 7, and 60 °C) and cooling (27 and −3 °C). The diffractogram at the top (cap.@-3 °C) was obtained (in transmission) from a pure powder sample within a glass capillary and used as a reference. Vertical tick marks highlight the C_10_*P*2_1_/*c* reflections as inferred from Rietveld refinement (*h*00 reflections in black; *hkl* with *k* and/or *l*≠0 in red). Data for PDMS C12 and PDMS C14 are shown in SI (Figures SI3 and SI4).

Grazing-incidence synchrotron X-ray powder diffraction (GIXRD) was employed, *in situ*, to investigate structural features of pure and encapsulated decanoic, lauric, and myristic acids at different temperatures on the MCX beamline of the Elettra synchrotron light source (Trieste, Italy).^66^ For each fatty acid, a reference diffractogram was acquired using a pure powder sample inside a spinning glass capillary, collected in a transmission geometry at −3 °C with the Oxford Instruments cryojet. On the reference fatty acid samples, all peaks are well-indexed by the *hkl* reflections expected for these molecules crystallizing in the *P*2_1_/*c* space group (Figures 5, SI3, and SI4). It is worth highlighting the dominant intensity of the *h*00 reflection relative to planes parallel to the layers formed by packed fatty acids and the high intensity of the reflection related to the planes parallel to the molecular long axis (211 for decanoic and lauric acids and 311̅ in the case of myristic acid). In contrast, the PDMS-encapsulated fatty acids display only dominant contributions from the *h*00 reflections and a very weak 211 peak for decanoic and lauric acids (see Figures 5 and SI3) and a 311̅ peak for the myristic acid (Figure SI4). These results suggest the presence of some degree of structural disorder within the aliphatic layers in the PDMS-encapsulated samples, confirming our interpretation of the DSC data discussed above.

GIXRD analysis at different temperatures also allowed structural characterization of the encapsulated samples below and above the melting point of each fatty acid. As expected, above the melting points determined by DSC (Tables 2, SI2 and Figures S12–SI1), the diffraction peaks of the fatty acids disappeared and reappeared again during the subsequent cooling step, confirming the formation of a crystalline phase at the end of the thermal cycle.

**Figure 6:**
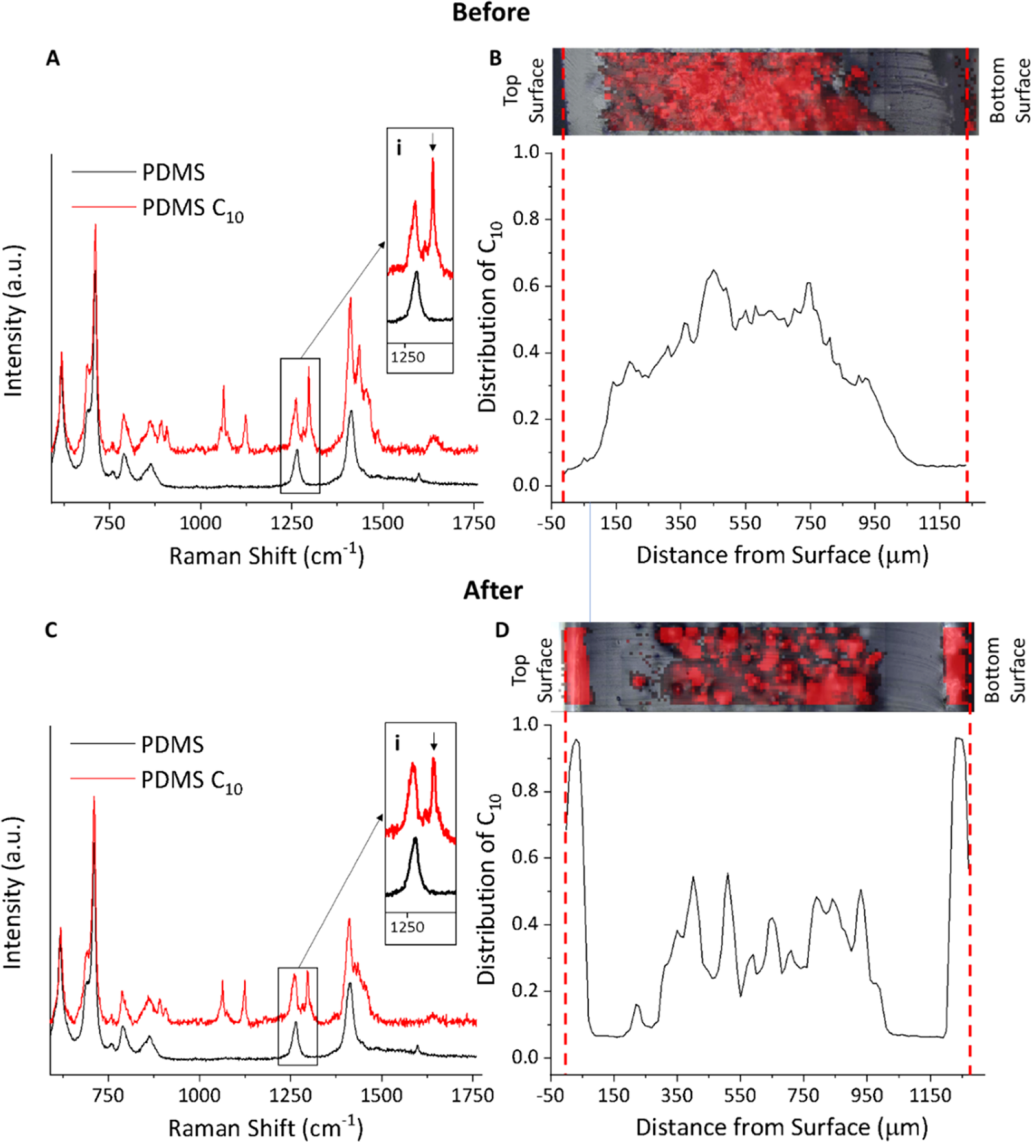
Mapping the delivery of cargo distribution for C_10_ following the temperature jump. Raman cross sections displaying the spectra (A and C) and the redistribution of decanoic acid within the PDMS matrix (B and D), before (A and B) and after (C and D) thermal response.

Finally, the distribution of the active cargo along the cross section of the encapsulated samples, before and after thermal activation, was followed by spatially resolved Raman spectroscopy. As discussed, the fatty acids were initially located within the encapsulation matrix, several tens of microns away from the surface (Figure 6a,b for C10 and Figures SI5–6a,b for C12 and C14, respectively). After thermally triggering to the specific melting temperatures for each fatty acid, Raman data clearly show that the diffusion of the cargoes from within the encapsulation matrix toward the surface of the material has occurred, delivering a localized front near the surface region. (Figure 6c,d for C10 and Figure SI5–6c,d for C12 and C14, respectively). This response can be rationalized in terms of the preparation method, which leaves the cargoes trapped out of equilibrium in the solid state within the encapsulation matrix due to the fast thermal quenching and subsequent surface rinse-off with ethanol. When the temperature increases and the melting point is reached, the liquid cargo is released out of the PDMS matrix and diffuses to the surface, thus delivering a thermally controlled response for the encapsulation system. Following the thermal response, for all three fatty acids, the distribution of residual fatty acid across the PDMS cross section is not homogeneous (Figures 6, SI5, and SI6). Diffusion and transport of molecules through PDMS is determined by complex interplays involving enthalpic and entropic effects and multiple interactions.^68−70^ Specifically, there are significant differences between the diffusivity of isolated molecules and clusters. The former have high diffusivities, while the latter have low diffusion probability. Therefore, one can anticipate that fatty acid aggregates loaded in a high concentration within the PDMS would be largely immobile. Temperature-induced onset of diffusion would essentially arise from molecules being released at the outer boundary of the fatty acid reservoir within the bulk PDMS. These individual released molecules diffuse rapidly to the interface, explaining the uneven distribution of fatty acids after thermal release.

### pH and Temperature-Triggered Release of the Active Cargo into Liquid Media

In the previous section, we demonstrated that the encapsulated fatty acids show two levels of physical–chemical response, depending on both the temperature and pH of the environment. This dual response can be exploited to deliver smart functions associated with the controlled release of the antimicrobial cargo from the encapsulation matrix.

**Figure 7:**
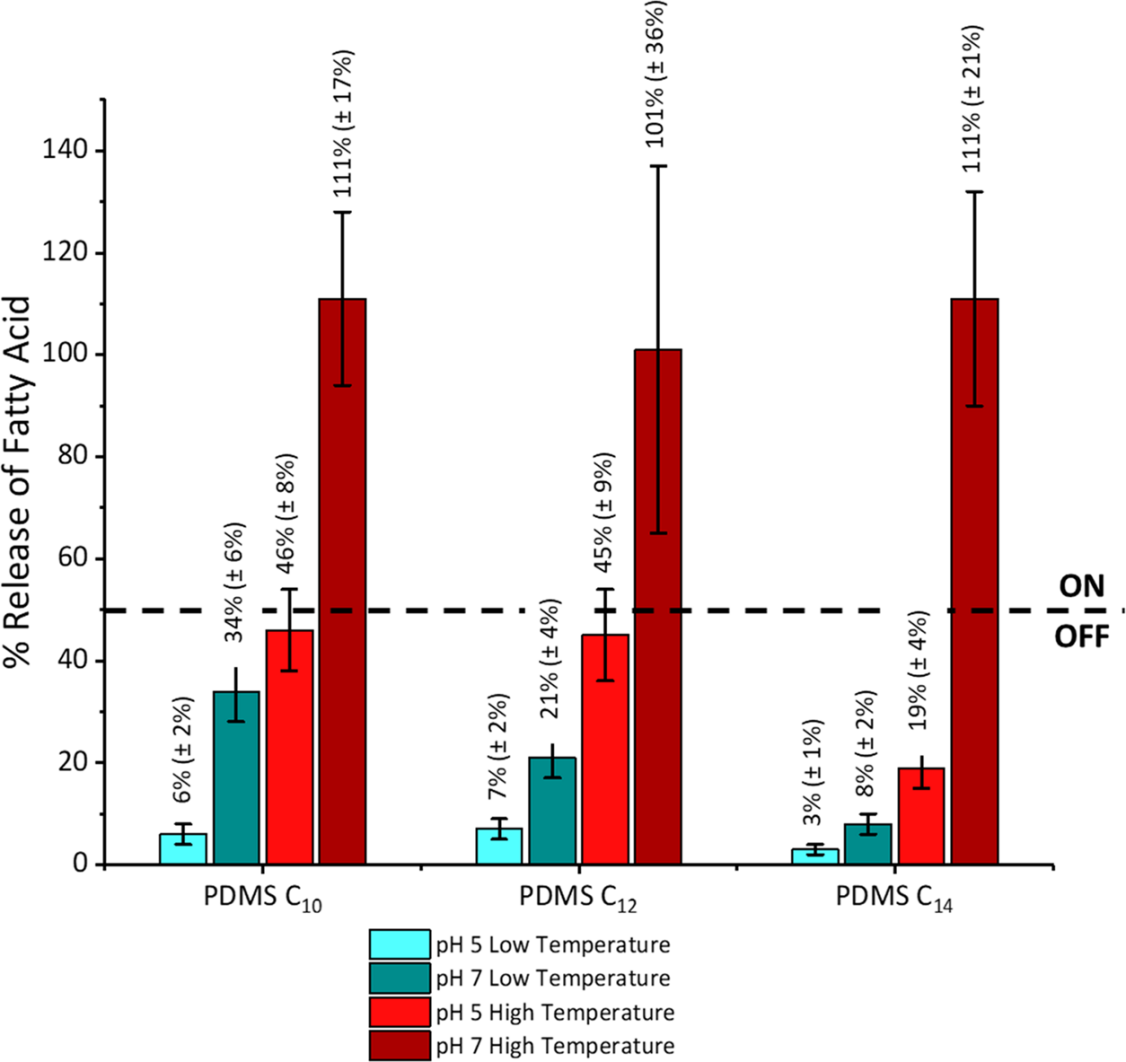
Fatty acid release into pH buffer solutions from PDMS surfaces loaded with decanoic (PDMS C_10_), lauric (PDMS C_12_), and myristic (PDMS C_14_) acids under different pH and temperature conditions. Release data were collected via gravimetric analysis before and after release; further details can be found in the SI. Release conditions are also described in the SI, briefly: temperatures for PDMS C10 samples, 5 and 37 °C; for PDMS C12 samples, at RT and 50 °C; for PDMS C14 samples, at RT and 60 °C.

First, the effect of pH and temperature control variables on the release of the cargoes in liquid environments was quantified using gravimetric analysis by weighing the samples before and after the release, with an average taken from eight samples for each experimental condition. The release experiments were carried out over a 24 h period. For these experiments, a 2^2^ factorial experimental design was used, with two levels of temperature (“low temperature” OFF, below the melting point and “high temperature” ON, above the melting point) and two levels of pH (pH 5 OFF and pH 7 ON) using buffer solutions as the liquid media in which the samples were placed. During the release experiments, we noticed a systematic bias on the determination that overestimated the mass of fatty acid released (release > 100%) but in all cases, this bias was smaller than the expanded uncertainties of the determinations (Figure 7). We can therefore consider this bias nonstatistically significant.

The release responses were evaluated gravimetrically and are summarized in Figure 7. The dotted line defines the ON and OFF binary states with respect to a threshold of 50% release. The value of 50% was selected based on a commonly accepted convention, used to transform a nondiscrete variable into a binary parameter, setting all cases below one-half of the maximum allowed value to OFF and all cases above one-half of the maximum allowed value to ON. The maximum release of the cargo was observed for temperatures above the melting point, where the cargo is in the liquid form, in combination with pH ≈ 7, when the solubility of the cargo in water is enhanced by deprotonation of the fatty acids. Conversely, minimal release occurs at low temperatures and acidic pH (pH ≈ 5) where the cargo remains in the solid form and the solubility of the protonated fatty acid is low. Residual releases are observed for the intermediate conditions, probably due to a compensation of temperature and solubility factors, but all have values below the threshold of 50% release that can be defined as the OFF state for a nondiscrete variable. The rationale behind our choice of 50% as the ON–OFF threshold will be further elaborated on in the following section devoted to biological assays.

Raman spectroscopy was used to investigate the cross sections of encapsulated acids in contact with the liquid media after the release experiments (Figures SI7–9). This data confirms that for the low pH and low temperature (switch-OFF conditions), the fatty acids remained mainly within the encapsulation matrix several tens of microns away from the surface. Conversely, for high pH and high temperature (switch-ON conditions), only a residual amount of fatty acid remains, either within the cross section or at the surface of PDMS, suggesting that most of the cargo has been released to the surrounding media. The intermediate “OFF” conditions showed that a considerable amount of fatty acid continues to be retained within the encapsulation matrix, with some relocation of the cargoes within the cross sections, explaining the small residual release discussed above.

Overall, the system displays two levels of temperature responses (“low temperature” OFF, below the melting point and “high temperature” ON, above the melting point) and two levels of pH responses (pH 5 OFF and pH 7 ON). In the following section, it will be demonstrated that this combined pH- and temperature-triggered response can be translated into a proof-of-concept system that shows effective antimicrobial activity selectively targeting planktonic or surface-attached bacteria.

### pH and Temperature-Triggered Antimicrobial Activity on Planktonic and Sessile Bacteria

The pH and temperature-triggered antibacterial performance of the encapsulated systems was investigated using the model microorganism *Escherichia coli* (*E. coli*). Considering the specific thermal response of the three encapsulated systems, we chose decanoic acid PDMS-C_10_ surfaces for these proof-of-concept biological experiments. This system is responsive around physiological temperature, with a melting point for the encapsulated decanoic acid at 29.8 ± 0.4 °C. Below this temperature, the system will remain “frozen” in the encapsulated state, while above (*e.g*. at 37 °C), the encapsulated cargo will be released. Additionally, decanoic acid displays a large change in solubility with pH in aqueous media, reaching 18.551 g L^–1^ at pH = 7 (Table SI1).

On the other hand, decanoic acid is one of the most antimicrobial naturally occurring saturated fatty acids.^10^ The primary target seems to be the bacterial cell membranes through the detergent effect that can solubilize key membrane components and create transient or permanent pores that compromise the cell integrity. *E. coli* is sensitive to decanoic acid, with minimum inhibitory concentrations (MIC90) between 0.32 and 0.4 mg/mL (Figure SI15), which is nearly independent of the pH. Interestingly, the MIC90 value is well below the critical micelle concentrations reported in the literature for decanoic acid (≈7 mg/mL),^67^ suggesting that this bioactive molecule can inhibit bacterial growth without forming micellar aggregates.

**Figure 8:**
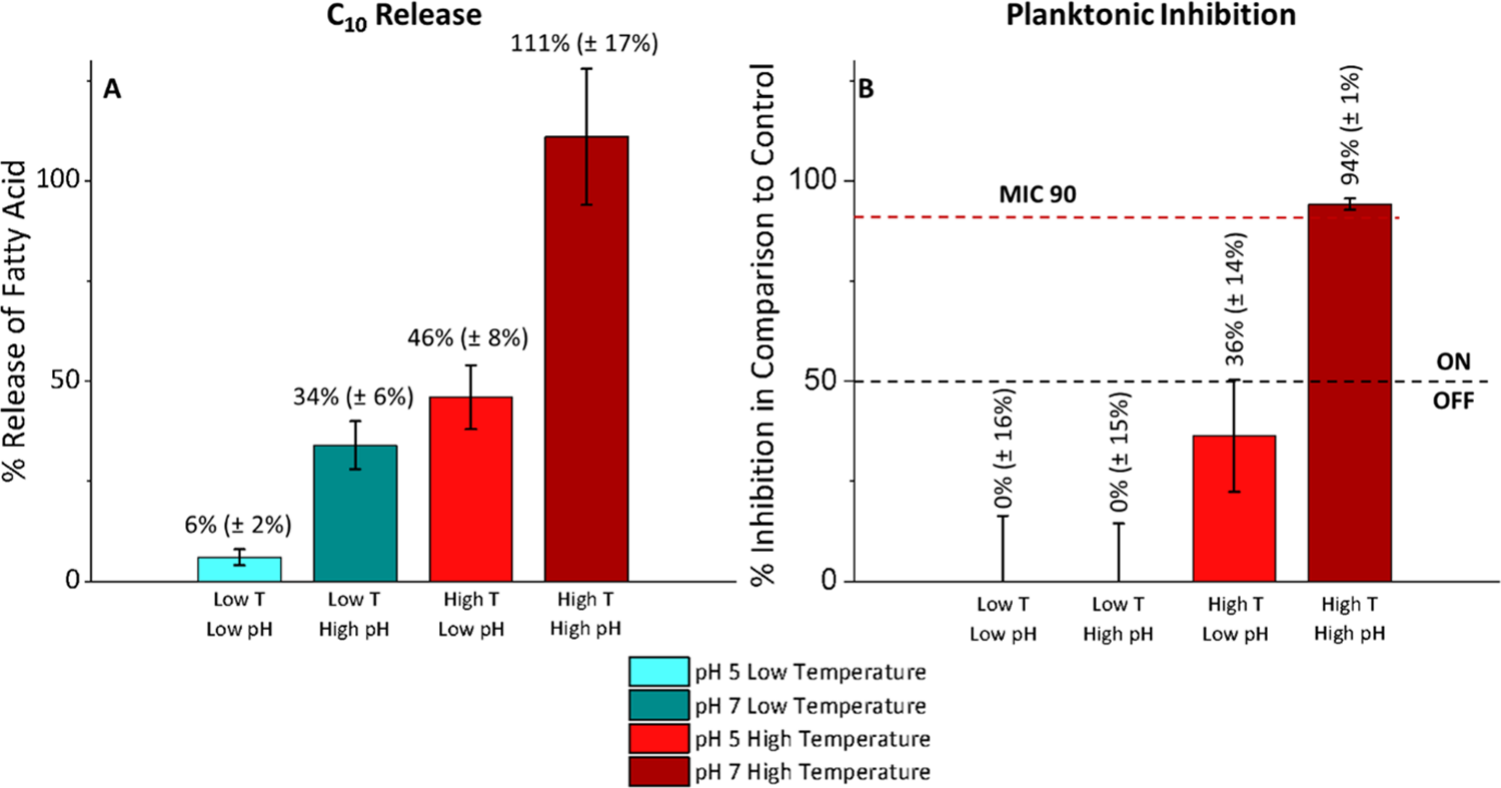
Correlating PDMS C_10_ release (A) and planktonic bacteria inhibition (B) of the C_10_-encapsulated system as a function of temperature and pH. The average values are inserted above each bar with the errors within brackets. In panel (B), the minimum inhibitory values at 90% (MIC90) are indicated by a horizontal line. Note that data shown in panel (A) have been already presented in Figure 7 for PDMS C10 samples. It has been reproduced here to aid direct comparison with planktonic inhibition data in panel (B). Temperature levels for PDMS C10 samples were 5 °C (low) and 37 °C (high).

First, planktonic bacteria were exposed to the encapsulated PDMS-C_10_ systems in liquid media. A good correlation between the microbial inhibition in the planktonic state and the release of fatty acid at specific combinations of the temperature and pH was observed (Figure 8a,b). The strongest inhibition for planktonic bacteria was observed when both temperature and pH variables were in the high value (ON) state, going over microbial inhibition at 50% (MIC50), which can be consistently considered the ON–OFF threshold value. As a matter of fact, the biological response in this case reached even the MIC90 (i.e., 90% inhibition) showing a net antimicrobial activity toward the planktonic state. For all of the other combinations of pH and temperature, the planktonic inhibition was below MIC50 and can be considered in the OFF binary state for the antimicrobial planktonic response, despite some quantitative difference observed for different pH and temperature combinations. The rational of the 50% ON–OFF threshold can then harmonize into a single binary framework two qualitatively different and nondiscrete parameters such as fatty acid release and microbial inhibition, as a result of a combination of low (OFF) and high (ON) control variable inputs for pH and temperature.

**Figure 9:**
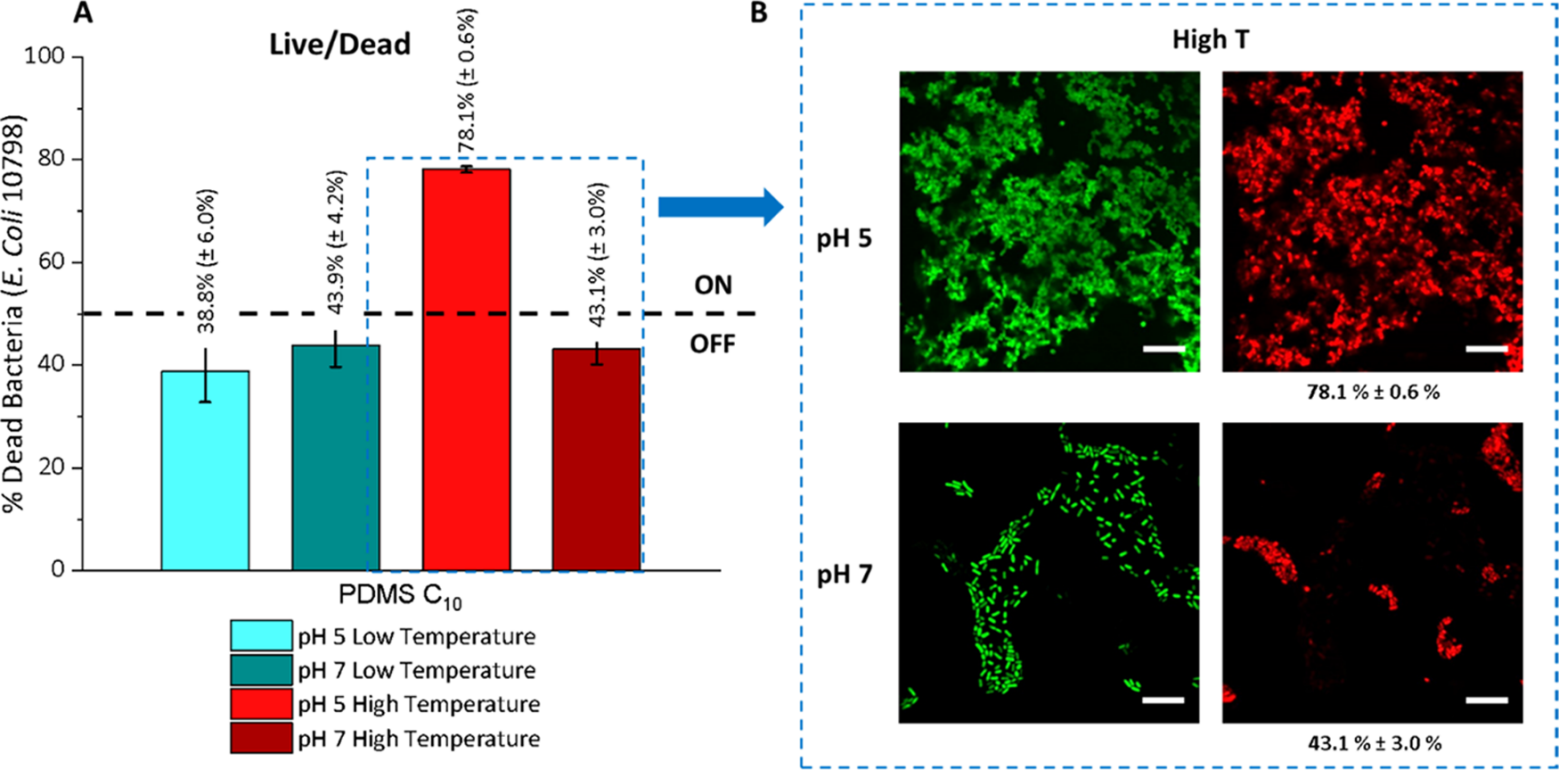
(A) Percentage of dead sessile bacteria (*E. coli* 10798) at the surface of PDMS C10 under different pH and temperature conditions, determined with Live/Dead staining. (B) Laser scanning microscopy micrographs of sessile bacteria under high-temperature conditions (37 °C) for both pH’s; the green channel is Syto9 unspecific staining of all cells, and red channel is propidium iodide selective staining of dead cells. Scale bars are 10 μm. Temperature levels for PDMS C10 samples were 5 °C (low) and 37 °C (high).

Second, the effect of pH and temperature on sessile *E. coli* cells was investigated for both the control PDMS and the encapsulated PDMS systems. The viability of the *E. coli* cells at the surface was evaluated using confocal laser scanning microscopy combined with Live/Dead staining. The PDMS controls showed no variation with temperature or pH, with significant populations of live sessile cells imaged at the surface (Figure SI16). However, the results shown in Figure 9 for the encapsulated system demonstrate that the maximum antimicrobial effect at the surface is obtained only for the combination of high temperature (ON) and low pH (OFF) states, while other parameter combinations (Figures 9 and SI17) showed a residual antimicrobial effect at the surface that is below the 50% threshold (OFF state).

These observations can be rationalized by considering the separate effects of the two control variables, temperature and pH, on the encapsulation system. The temperature trigger allowed the redistribution of the active cargo from the reservoir inside the polymeric matrix to the surface of the encapsulation system. Once at the surface, the pH determines whether the cargo remains at the surface (acidic pH) or is released into the liquid media (basic pH) by regulating the aqueous solubility of the fatty acid. Therefore, the combination of high temperature (ON) and low pH (OFF) ensures the maximum concentration of the fatty acid at the surface, delivering the most efficient antimicrobial effect on surface-attached bacteria.

Additionally, the surface coverage of bacteria also seems to be influenced by the overall response of the system, particularly at the high-temperature and high-pH state, attributed to the ON state for the planktonic state, which results in low viability of the planktonic bacteria due to the released C10 cargo. We note that a direct comparison between blank PDMS and active surfaces under the same experimental conditions enabled the antimicrobial effect to be normalized, thus compensating for any other intrinsic effects that may depend on temperature and pH, e.g., cell-surface adhesion dynamics.

### Proof of Concept toward Boolean Logic Gate Antimicrobial Systems

In the previous sections, we have demonstrated that by combining pH and temperature responses, it is possible to have a system that not only exhibits ON/OFF states but can also control relocation and targeting of the active cargo toward the surface or the liquid media, leading to different ON/OFF states for the planktonic and sessile *E. coli* bacteria. Our results provide a proof-of-concept platform that may enable the design of antimicrobial systems able to operate as logic gates, delivering different responses under different environmental conditions.

**Figure 10:**
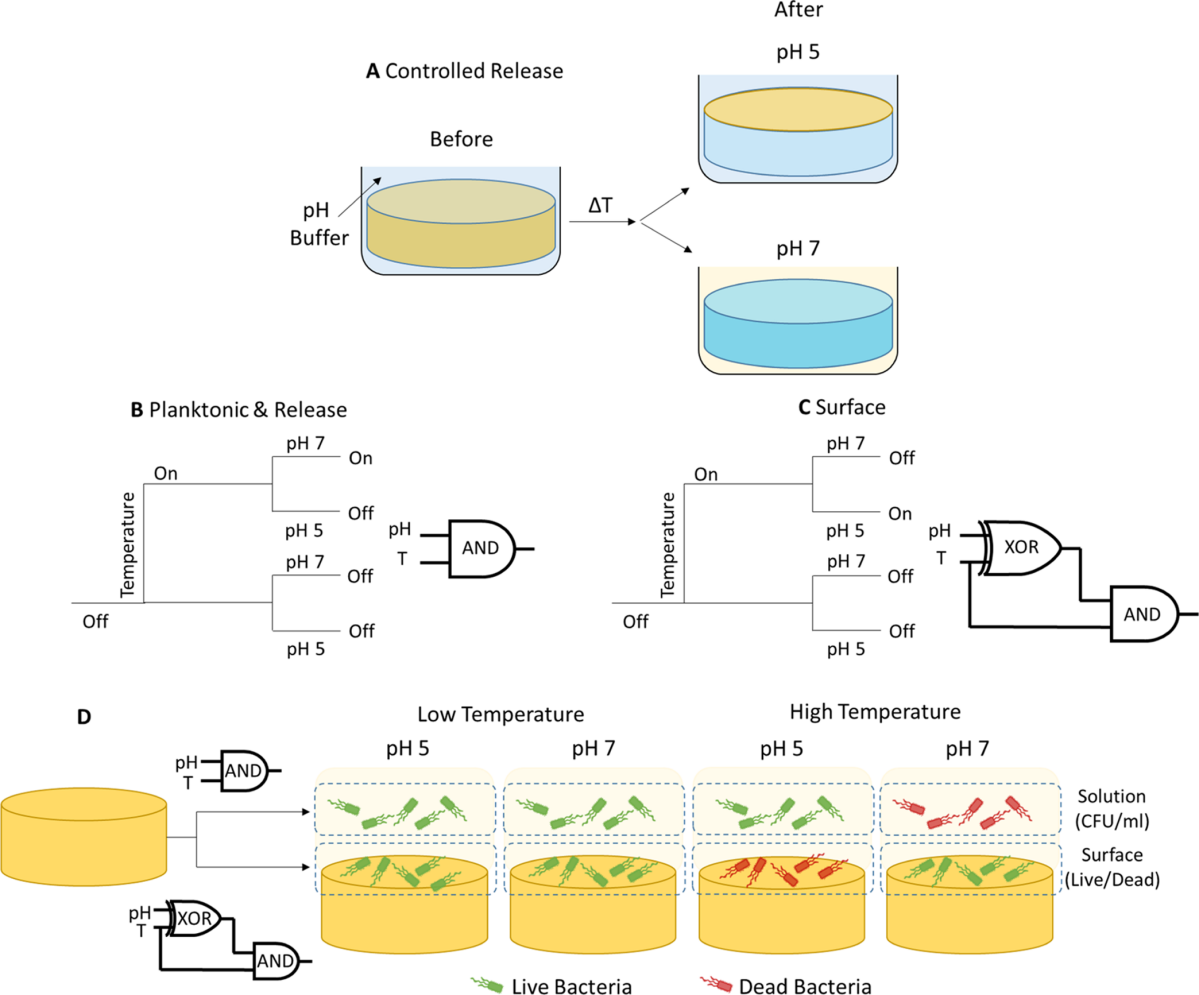
Design principle of the responsive systems resembling logic gate operations. (A) Temperature and pH-controlled release; (B) *E. coli* experiments, response into the planktonic media; (C) *E. coli* experiments, response at the surface; and (D) overall diagram showing the working principle of the system.

For example, our system delivers two distinct levels of response for *E. coli*: one into the liquid media and another one directly at the surface. In both cases, the temperature “gate” controls the extent of cargo released from within the encapsulation matrix, while the pH determines whether the cargo remains at the surface or is dispersed into the liquid media. These two processes can be rationalized in terms of a series of logic operations between the two input variables, temperature and pH (Figure 10). Translating the 2^2^ factorial experimental design into binary input signals (*e.g*. low OFF, high ON), we can associate the logical operator AND to the response delivered toward the planktonic state, occurring only when both control variables are in the ON state. Conversely, the effect delivered at the surface emerges as a combination of XOR and AND operations between the temperature and pH variables. The schematic description of this complex responsive system is illustrated in Figure 10.

We note that temperature and pH are important triggering signals for antimicrobial and antibiofilm technologies in medical applications, e.g., in stimuli-responsive wound dressings and catheters. Our system utilizes only the inherent physicochemical behavior of naturally occurring fatty acids within the widely used PDMS matrix to create a dual-responsive system that delivers location-specific antimicrobial responses. Although this work is just a proof-of-concept study, there are important aspects that provide a framework for designing a specific technology, e.g., for wound dressings or catheters. It is known that infections in wounds^71,72^ and in catheter-associated urinary tract infections^73,74^ progress through various stages. At the early stages, it is bacterial contamination and colonization by early-stage biofilms at the surface of the wound/skin or catheter that needs to be addressed. In such conditions, when there is no bodily response to the wound, the normal temperature of the skin will release the encapsulated fatty acid from the matrix, while the acidic pH of the environment (e.g., healthy skin pH values around 5 and 5.5) would mean that the AND/XOR logic gate applies and the antimicrobial agent would be located at the device surface where the problem needs addressing. When a wound or catheter infection develops further into critical colonization, the proliferating bacteria and infection extend into the surrounding areas. At this stage, there is a bodily response to the developing infection, and the wound/catheter environment develops a more basic pH, often accompanied by a bodily temperature increase in the presence of infection. In such a case, the AND logic gate would operate and trigger the release of the biocide out of the carrier matrix and into the surrounding environment. Finally, it is important that the encapsulated material is retained within the PDMS matrix at storage temperatures that are lower than the operating body temperature; i.e., the system is in the OFF state.

## Conclusions

A dual stimuli-responsive system that is capable of location-specific cargo release has been successfully created by encapsulating a bioactive cargo of saturated fatty acids into a biocompatible inert polymeric matrix of PDMS. The thermal and pH responsivity of the system is delivered by the phase transition and solubility response of the active component itself, with the PDMS encapsulation matrix acting as a carrier and physical barrier to prevent passive release of the cargo into the media.

Spatially resolved Raman spectroscopy and thermal and structural material analysis have enabled the system behavior and response to be mapped. These data show that the system responds to two control variables, temperature and pH, which determines whether the active cargo relocates toward the surface or is released in the liquid media. Our exemplar system shows that two levels of antimicrobial response are achieved for *E. coli* under distinct combinations of stimuli: one response toward the planktonic media and another response directly at the surface for sessile bacteria. The system behavior resembles that of Boolean logic gates (e.g., low OFF, high ON). Thus, we can associate the logical operator AND to the antimicrobial response delivered toward the planktonic state, occurring only when both control variables are in the ON state. Conversely, the antimicrobial effect delivered at the surface arises from a combination of XOR and AND operations between the temperature and pH variables.

The approach proposed herein is technologically simple and scalable, facing low regulatory barriers within food and healthcare sectors by using approved components and relying on fundamental chemical processes. Our results also provide a proof-of-concept platform for the design and easy fabrication of antimicrobial systems capable of operating as logic gates, delivering different responses under different environmental conditions.

## Supporting Information Available

Experimental details and methods, relevant properties of fatty acids, DSC data, additional GIXRD data, additional Raman cross-sectional data, Raman histograms of component fit analysis, MIC values, and additional fluorescence microscopy images (PDF)

## Abbreviations

PDMS: polydimethylsiloxane
DSC: differential scanning calorimetry
GIXRD: grazing-incidence synchrotron X-ray powder diffraction
C10: decanoic acid
C12: lauric acid
C14: myristic acid
p*K*_a_: protonation equilibrium constant
E. coli: *Escherichia coli*
MIC90: minimum inhibitory concentrations at 90%
MIC50: minimum inhibitory concentrations at 50%
