# Supplementary material for: Temperature and pH Stimuli-Responsive System Delivers Location-Specific Antimicrobial Activity with Natural Products
Source: ACS Appl Bio Mater. 2023 Dec 11;7(1):131–43. doi: 10.1021/acsabm.3c00588 (PMC10792665; doi:10.1021/acsabm.3c00588)
Supplement: Supplementary file 1 — mt3c00588_si_001.pdf [file mt3c00588_si_001.pdf]

SUPPORTING INFORMATION

Temperature and pH stimuli-responsive system delivers location-specific antimicrobial activity with natural products.

Gareth Morris,[1,4] Sean Goodman,[1] Ioritz Sorzabal Bellido,[1,†] Chiara Milanese,[2] Alessandro Girella,[2] Piersandro Pallavicini,[2] Angelo Taglietti,[2] Mattia Gaboardi,[3] Frank Jäckel,[4] Yuri A. Diaz Fernandez,[1;2;],Rasmita Raval[1;**].*

[1]Open Innovation Hub for Antimicrobial Surfaces, Surface Science Research Centre, University of Liverpool, L69 3BX, UK

E-mail: *ydf@unipv.it, **raval@liverpool.ac.uk

[2] Department of Chemistry, University of Pavia, Via Taramelli 12, Pavia, 27100, Italy

[3] Materials Physics Center, CSIC-UPV/ EHU, Donostia - San Sebastian 20018, Spain

[4] Department of Physics and Stephenson Institute for Renewable Energy, University of Liverpool, L69 7ZE, UK

Table SI1. Relevant physical and chemical properties of natural fatty acids.

Fatty Acid	Carbon Chain Length	Literature Melting Point (°C)[1]	Literature pKa[2,3]	Aqueous Solubility (g L^{-1})	
				Fatty Acid (COOH)[4,5]	Fatty Acid Salt (COO^-)[2,6,7]
Caproic Acid	6	-4.1	4.83	10.2	138.14
Octanoic Acid	8	16.51	4.89	0.8	58.336
Decanoic Acid	10	31.39	4.9	0.15	18.551
Lauric Acid	12	43.82	5.3	0.055	5.113
Myristic Acid	14	54.16	5.8	0.02	1.727
Palmitic Acid	16	62.49	8.6	0.0072	0.473
Stearic Acid	18	69.3	10.15	0.0029	0.306

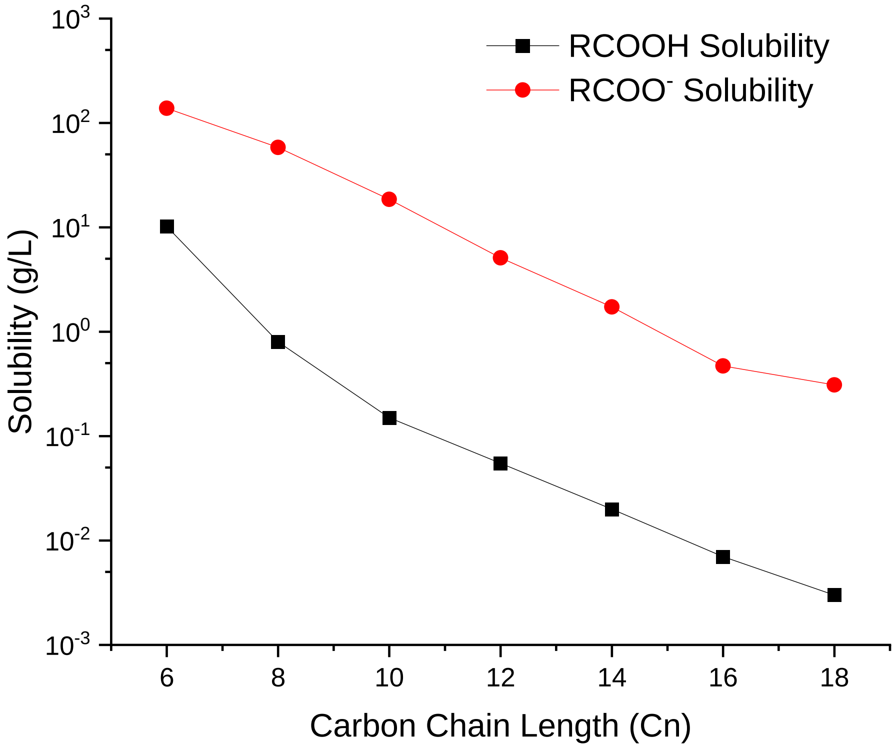

Figure SI1. Water solubility of natural fatty acids at acidic[4] (black) and basic[2,6,7] (red) pH as a function of aliphatic chain length. Cn is the total number of carbons. R refers to linear aliphatic chain Data presented here are in logarithmic scale for comparison to linear scale in Figure 1 in the main text.

Table SI2. Differential scanning calorimetry (DSC) data for pure and PDMS-encapsulated decanoic, lauric, and myristic acids, including calculated enthalpy values not shown in the main text. ΔH_{melt} data calculated combining the DSC overall enthalpy for the loaded samples (heat per g of the acid + polymer sample) with the average loading of the different fatty acids in the polymer obtained by gravimetric methods.

	**Sample type**	**Average T_{melt} (°C)**	**Average ΔH_{melt} (J/g of fatty acid)**
Decanoic (C_{10})	pure	32.7 (±0.1)	157 (±3)
	encapsulated	29.8 (±0.4)	248 (±29)
Lauric (C_{12})	pure	45.2 (±0.3)	180 (±4)
	encapsulated	37.6 (±1.7)	338 (±34)
Myristic (C_{14})	pure	55.4 (±0.7)	190 (±1)
	encapsulated	45.7 (±3.6)	328 (±114)

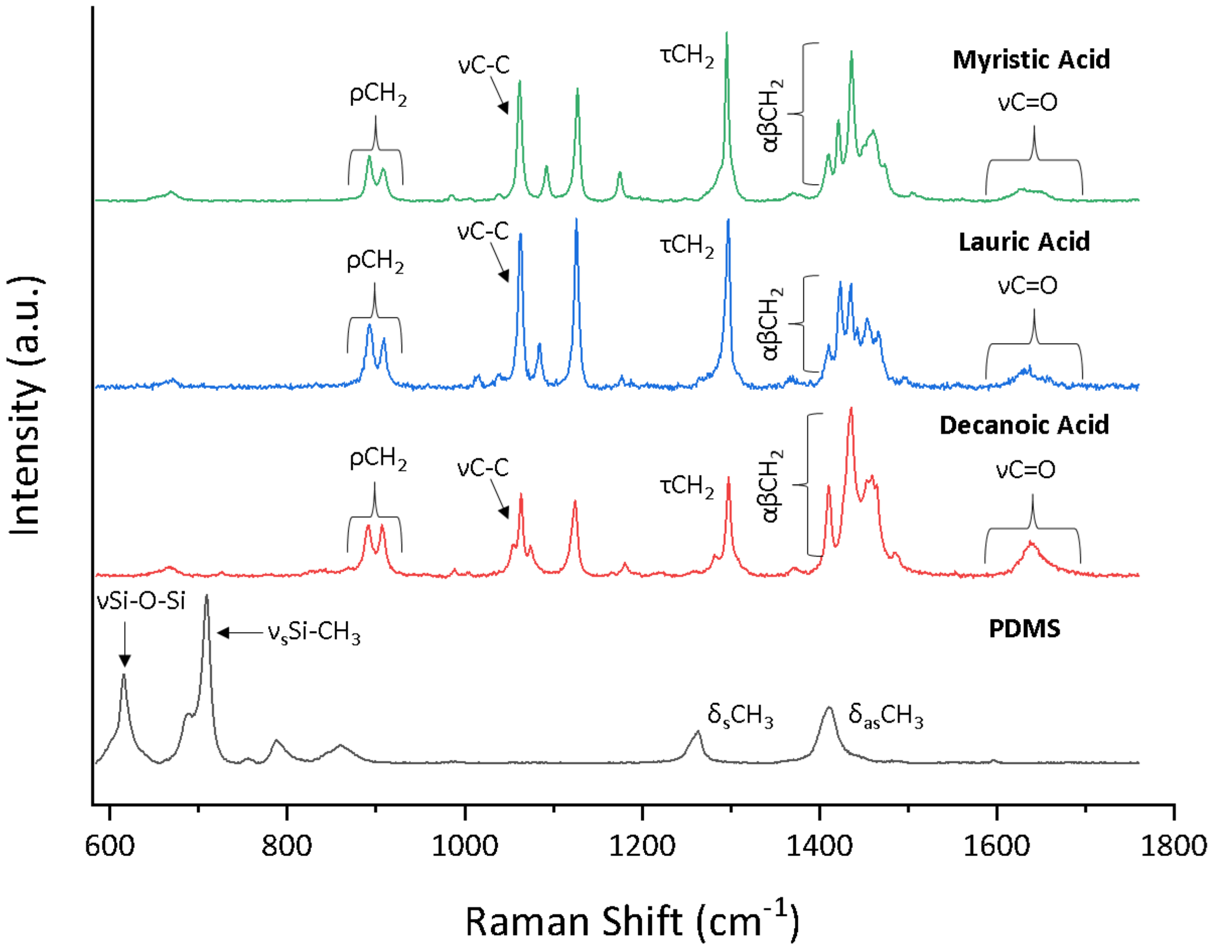

Figure SI2. Typical Raman spectra for pristine PDMS, decanoic acid, lauric acid, and myristic acid.

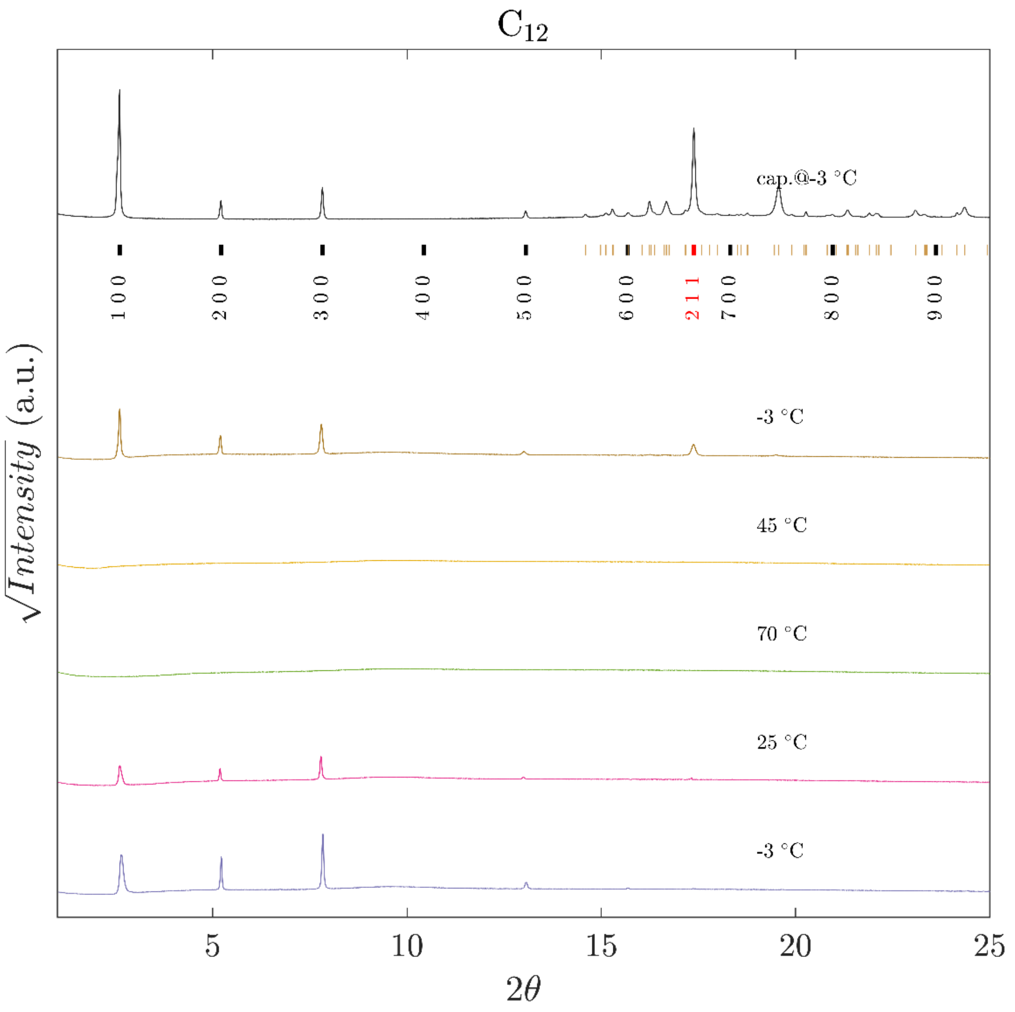

Figure SI3. Grazing-incidence synchrotron X-ray powder diffraction (GIXRD) of lauric acid encapsulated PDMS samples (PDMS C_{12}) at different temperatures. The diffractogram at the top (cap.@-3°C) was obtained from a pure powder sample within a glass capillary and used as a reference.

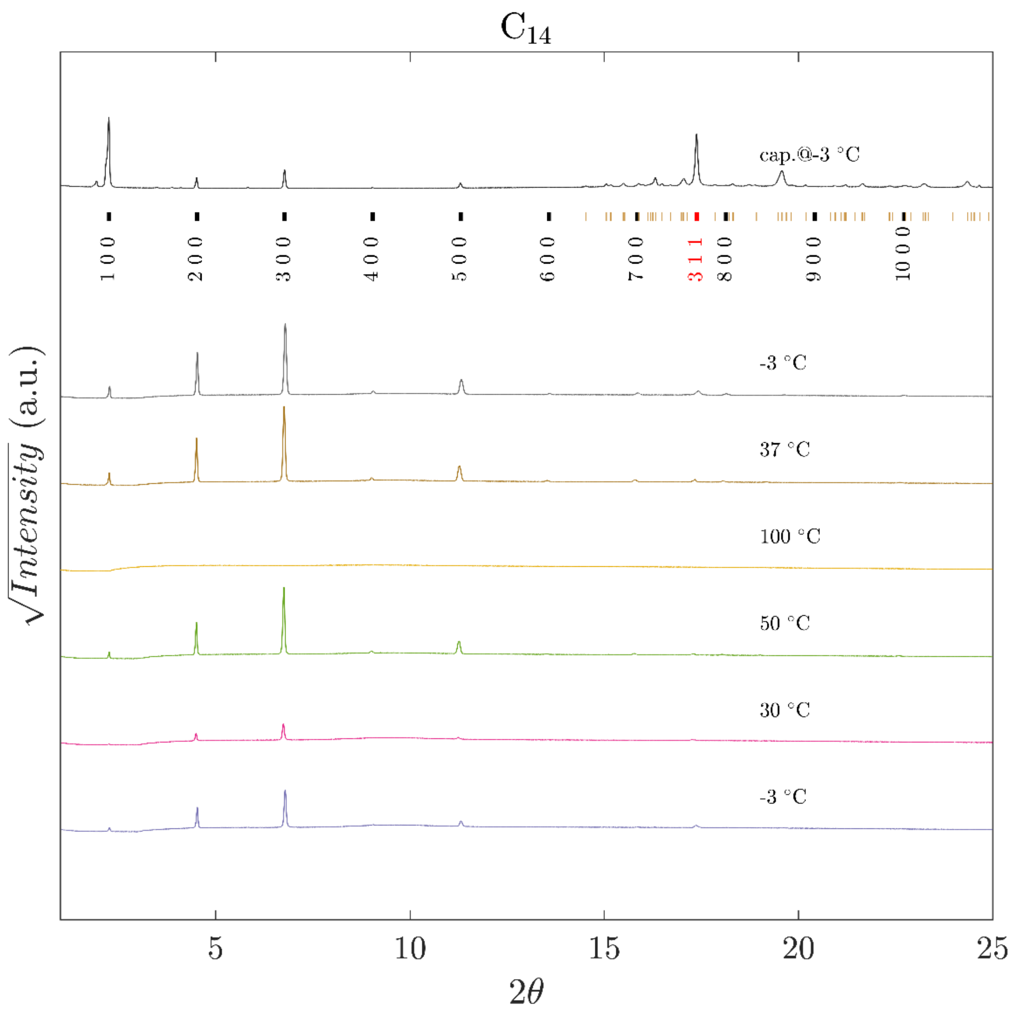

Figure SI4. Grazing-incidence synchrotron X-ray powder diffraction (GIXRD) of myristic acid encapsulated PDMS samples (PDMS C_{14}) at different temperatures. The diffractogram at the top (cap.@-3°C) was obtained from a pure powder sample within a glass capillary and used as a reference.

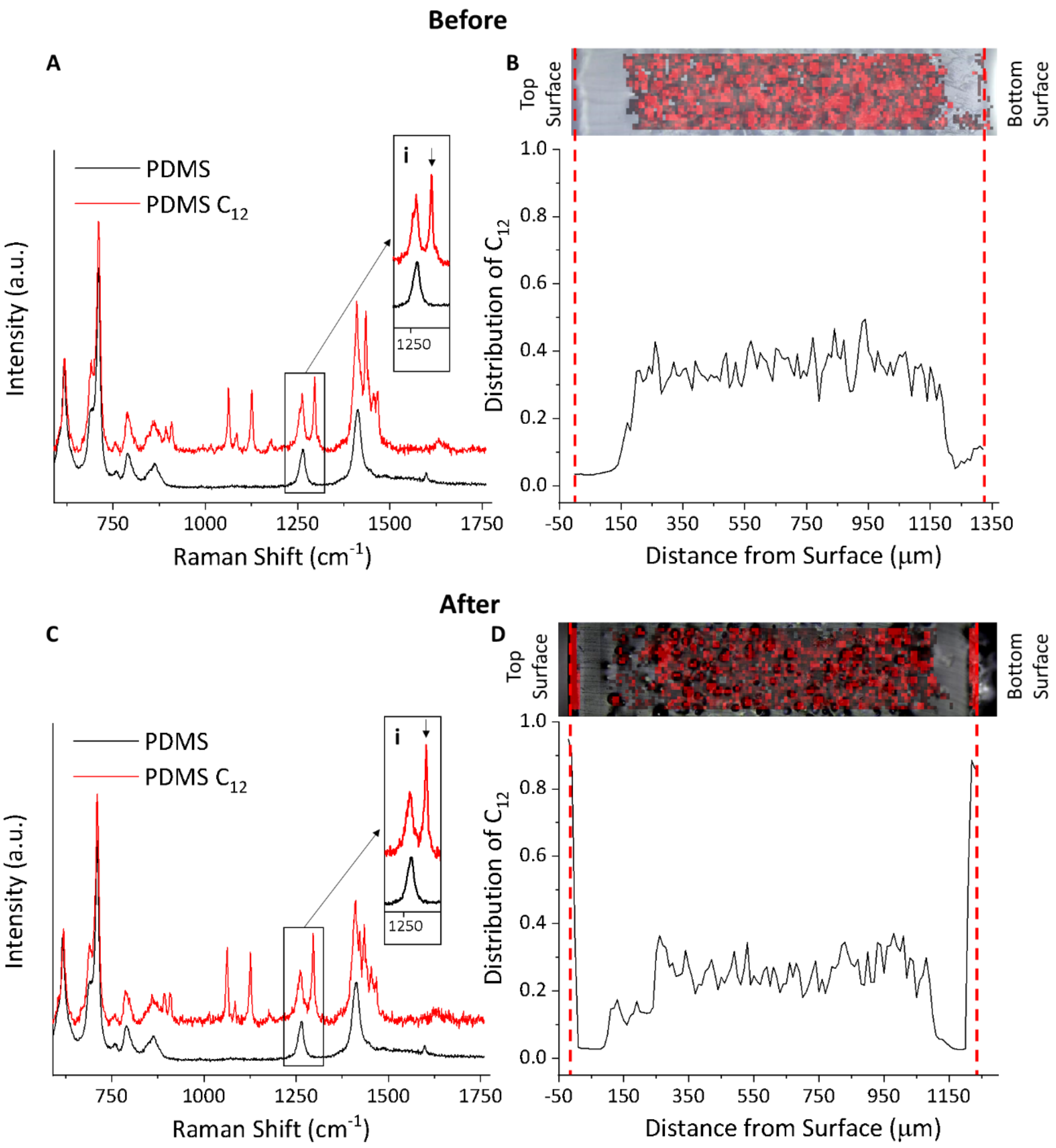

Figure SI5. Raman cross sections displaying the spectra (A and C) and the re-distribution of lauric acid within the PDMS matrix (B and D) as before (A and B) and after (C and D) thermal response. (i) Insert showing τCH_2 twist vibration[8] of Lauric acid.

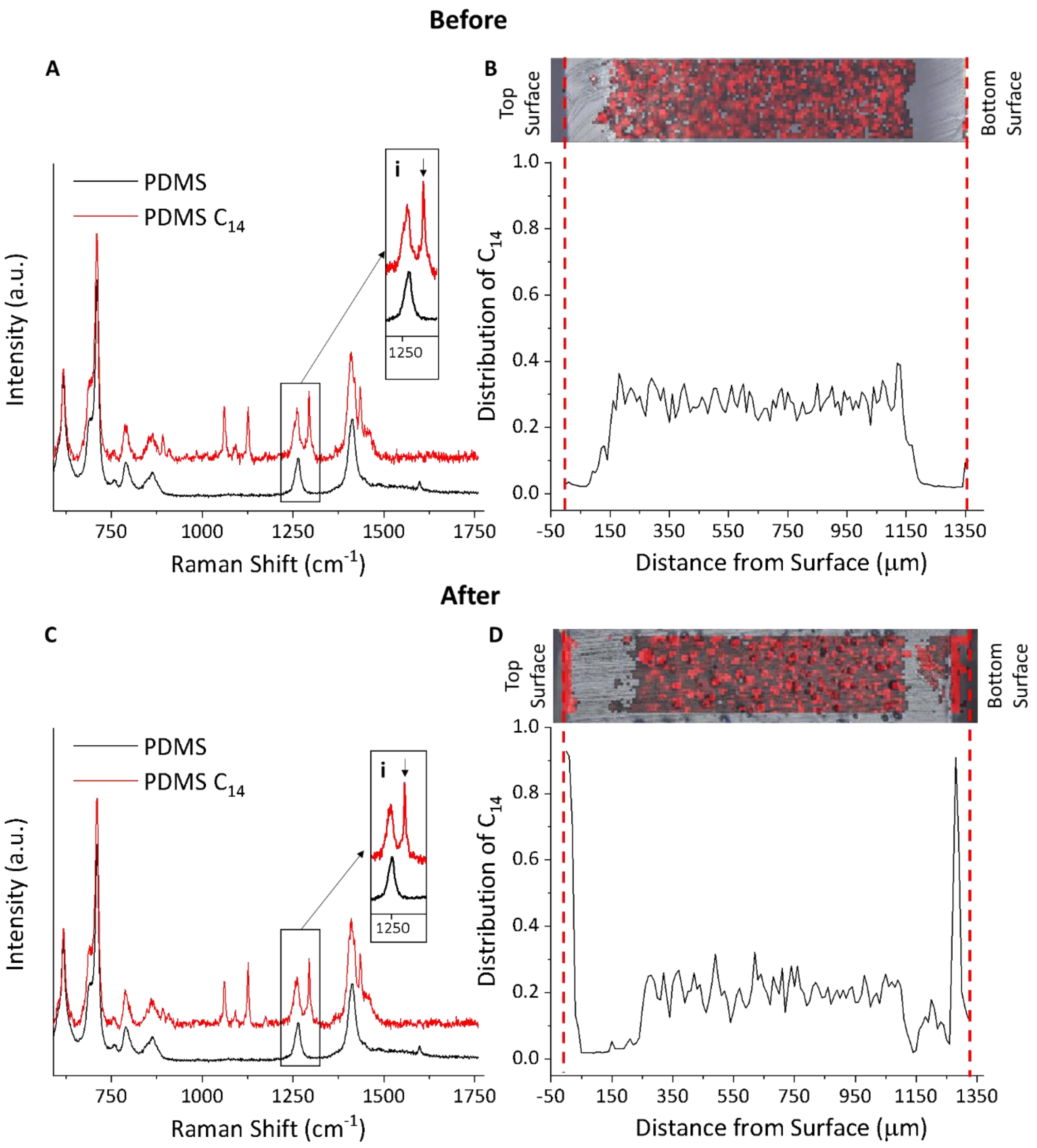

Figure SI6. Raman cross sections displaying the spectra (A and C) and the re-distribution of myristic acid within the PDMS matrix (B and D) as before (A and B) and after (C and D) thermal response. (i) Insert showing τCH_2 twist vibration[8] of Myristic acid.

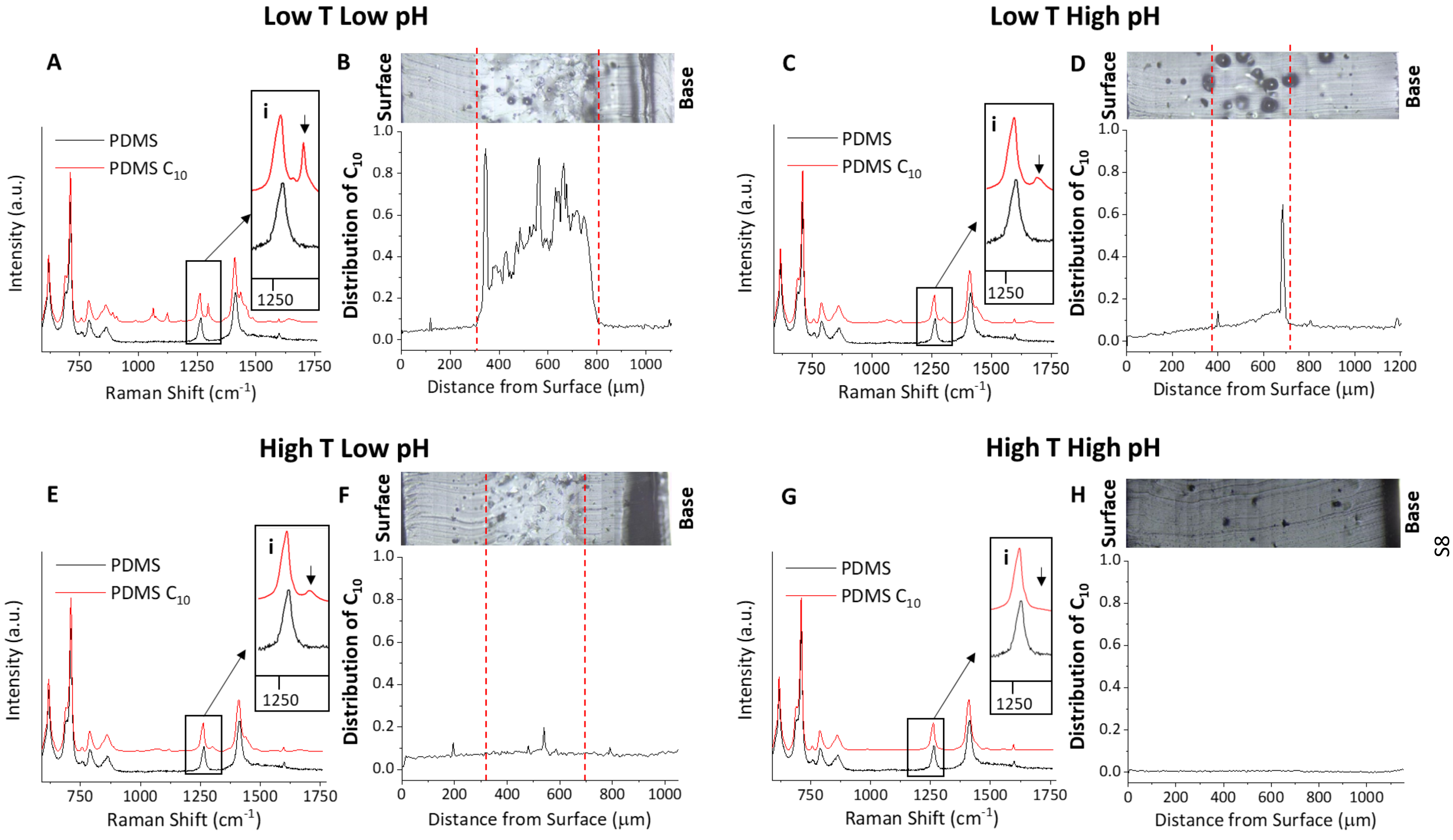

Figure SI7. Representative Raman data for PDMS C_{10} after each release condition. (A and B) Low temperature, low pH release (A) Representative Raman spectra of PDMS (black) & PDMS C_{10} (red), (B) Distribution of decanoic acid across PDMS C_{10}. (C and D) Low temperature, high pH release (C) Representative Raman spectra of PDMS (black) & PDMS C_{10} (red), (D) Distribution of decanoic acid across PDMS C_{10}. (E and F) High temperature, low pH release (E) Representative Raman spectra of PDMS (black) & PDMS C_{10} (red), (F) Distribution of decanoic acid across PDMS C_{10}. (G and H) High temperature, high pH release (G) Representative Raman spectra of PDMS (black) & PDMS C_{10} (red), (H) Distribution of decanoic acid across PDMS C_{10}. (i) Insert showing τCH_2 twist vibration[7] of decanoic acid for each release condition.

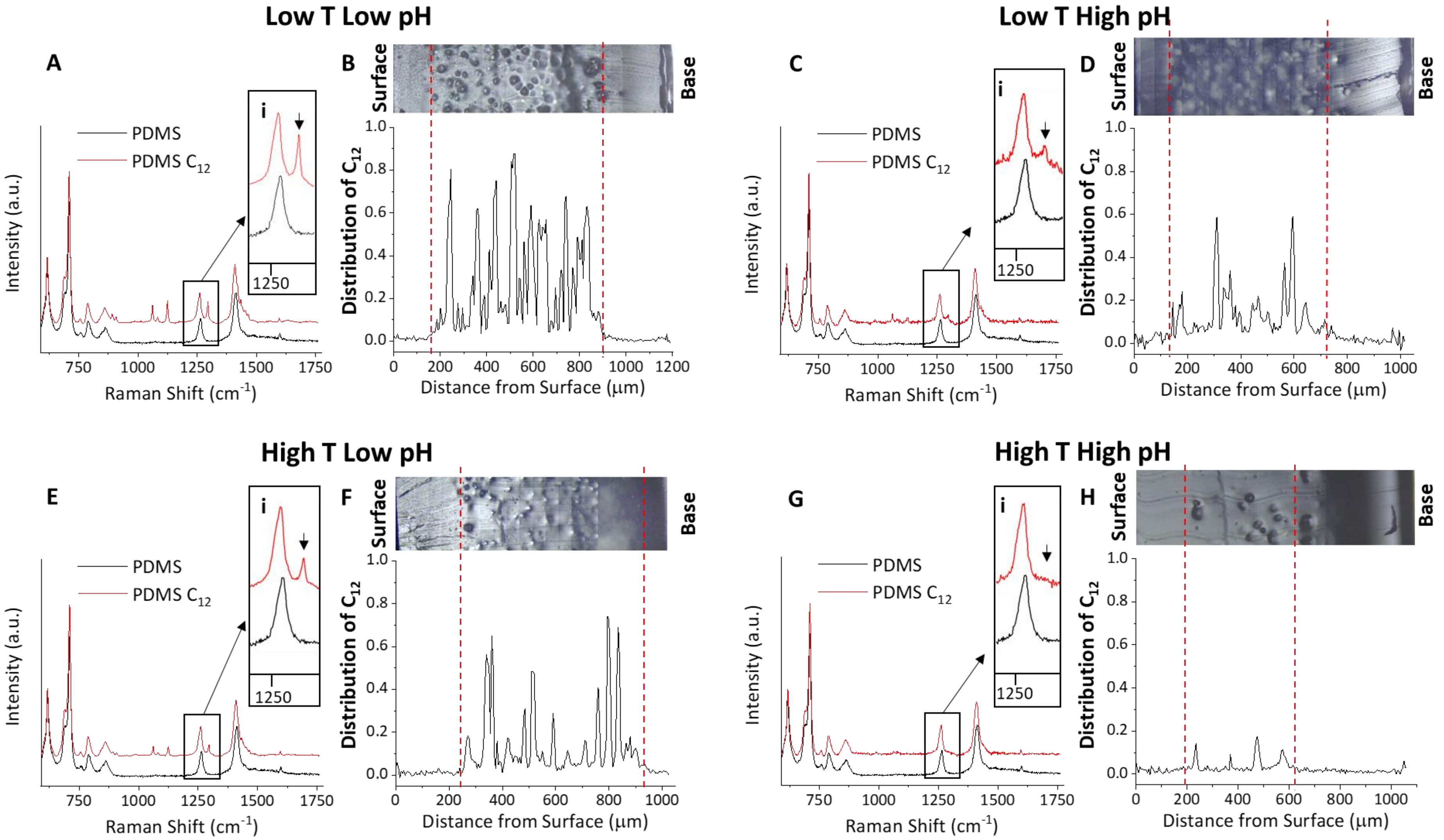

Figure SI8. Representative Raman data for PDMS C_{12} after 24h in each release condition. (A and B) Low temperature, low pH release (A) Representative Raman spectra of PDMS (black) & PDMS C_{12} (red), (B) Distribution of lauric acid across PDMS C_{12}. (C and D) Low temperature, high pH release (C) Representative Raman spectra of PDMS (black) & PDMS C_{12} (red), (D) Distribution of lauric acid across PDMS C_{12}. (E and F) High temperature, low pH release (E) Representative Raman spectra of PDMS (black) & PDMS C_{12} (red), (F) Distribution of lauric acid across PDMS C_{12}. (G and H) High temperature, high pH release (G) Representative Raman spectra of PDMS (black) & PDMS C_{12} (red), (H) Distribution of lauric acid across PDMS C_{12}. (i) Insert showing τCH_2 twist vibration[7] of lauric acid for each release condition.

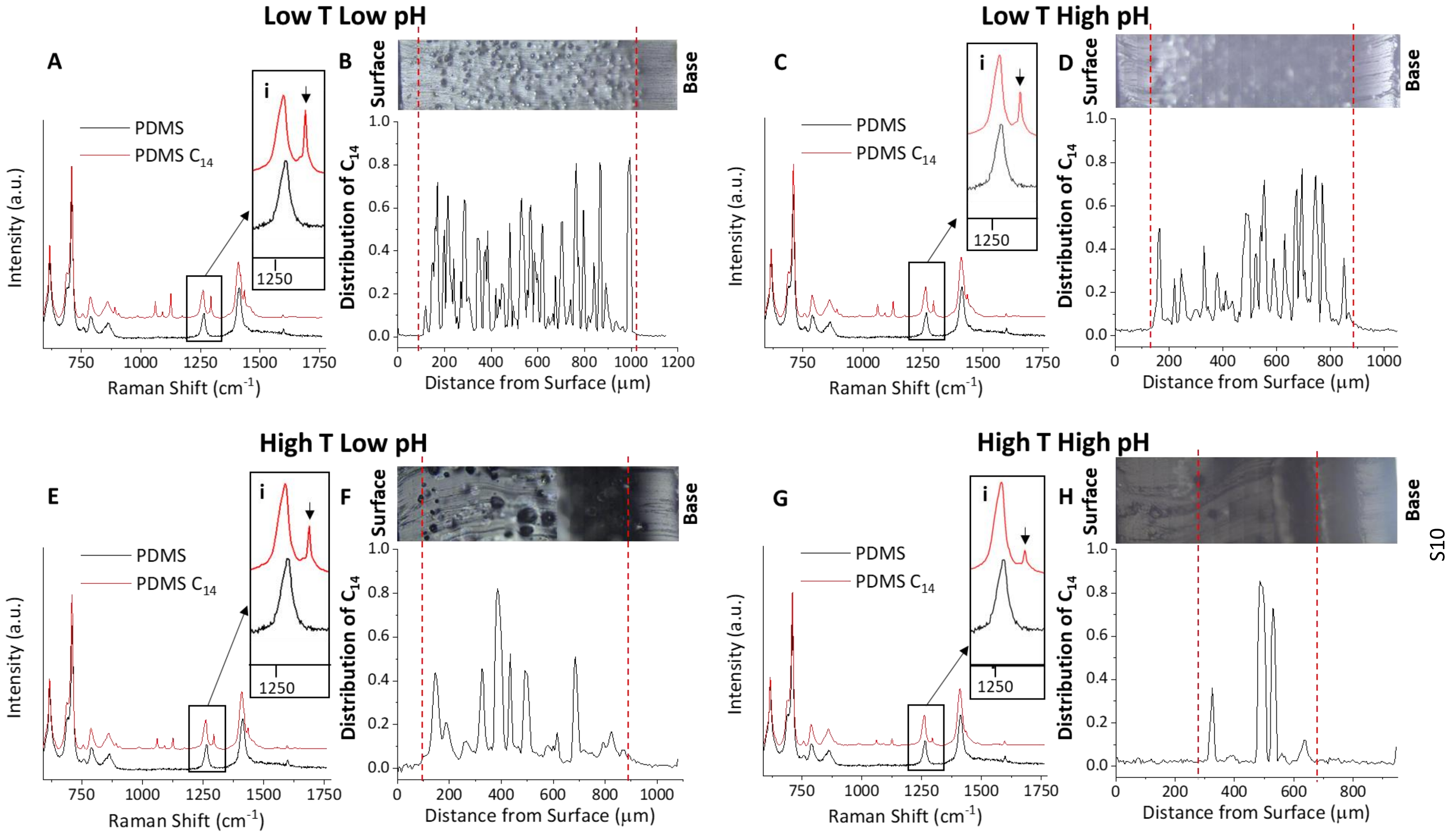

Figure SI9. Representative Raman data for PDMS C_{14} after 24h in each release condition. (A and B) Low temperature, low pH release (A) Representative Raman spectra of PDMS (black) & PDMS C_{14} (red), (B) Distribution of myristic acid across PDMS C_{14}. (C and D) Low temperature, high pH release (C) Representative Raman spectra of PDMS (black) & PDMS C_{14} (red), (D) Distribution of myristic acid across PDMS C_{14}. (E and F) High temperature, low pH release (E) Representative Raman spectra of PDMS (black) & PDMS C_{14} (red), (F) Distribution of myristic acid across PDMS C_{14}. (G and H) High temperature, high pH release (G) Representative Raman spectra of PDMS (black) & PDMS C_{14} (red), (H) Distribution of myristic acid across PDMS C_{14}. (i) Insert showing τCH_2 twist vibration of myristic acid[7] for each release condition

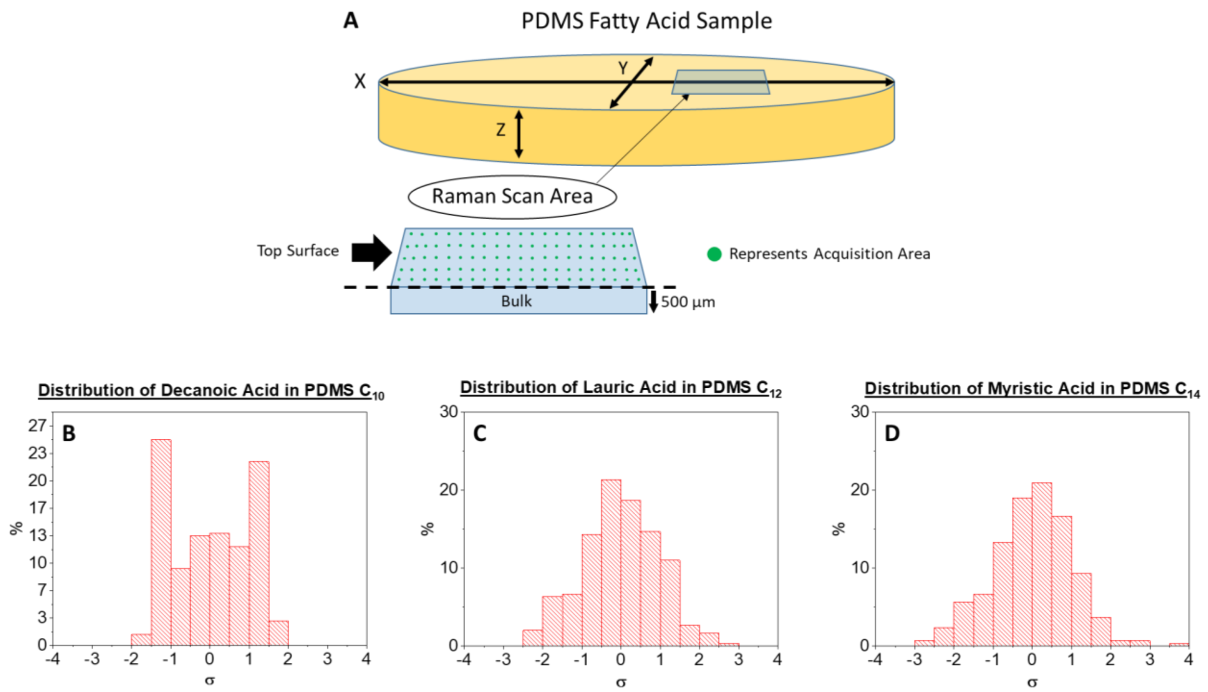

Figure SI10. Normalised histograms showing the component fit analysis[9,10] of Decanoic, Lauric and Myristic acid distributed across encapsulated PDMS samples, 500 µm below the top surface. (A) Schematic representation of the Raman experiment showing 1500 µm x 375 µm scan area (not to scale): green dots represent the area of acquisition across the surface of the sample.

Data from C_{12} and C_{14} is discussed in detail in the main text. For the encapsulated decanoic acid (C_{10}), we also observed a narrow intensity distribution with 90% of the data points were located within two standard deviations from the mean (Figure SI10 B). However, the distribution is bimodal indicating the presence of local areas with slightly higher and lower concentrations of the active compound. It is important to note that Raman spectra were collected at room temperature, which for decanoic acid is relatively close to the melting point of the encapsulated material and, therefore, the distribution will be affected by self-diffusion within the matrix.

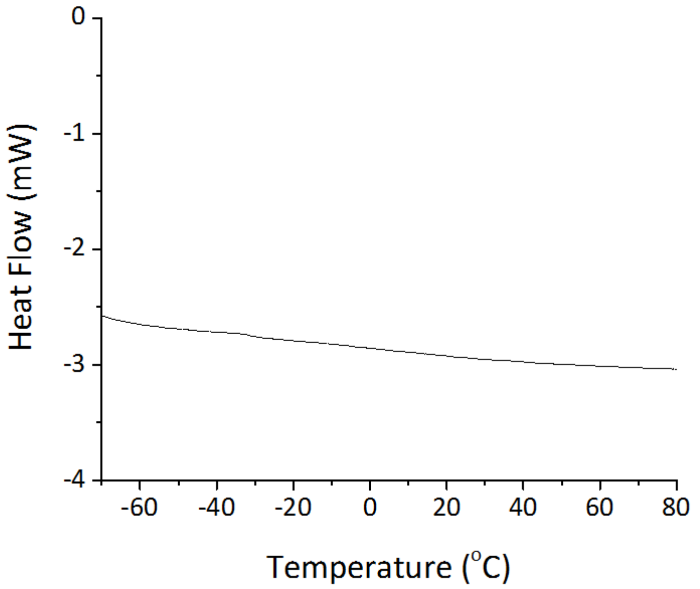

Figure SI11. Differential scanning calorimetry (DSC) profile for pristine PDMS, showing no thermal transitions between -70 ºC to +80 ºC. (Exo – up). Please note that this temperature range is well above the glass transition of PDMS (≈-123 ºC).[16]

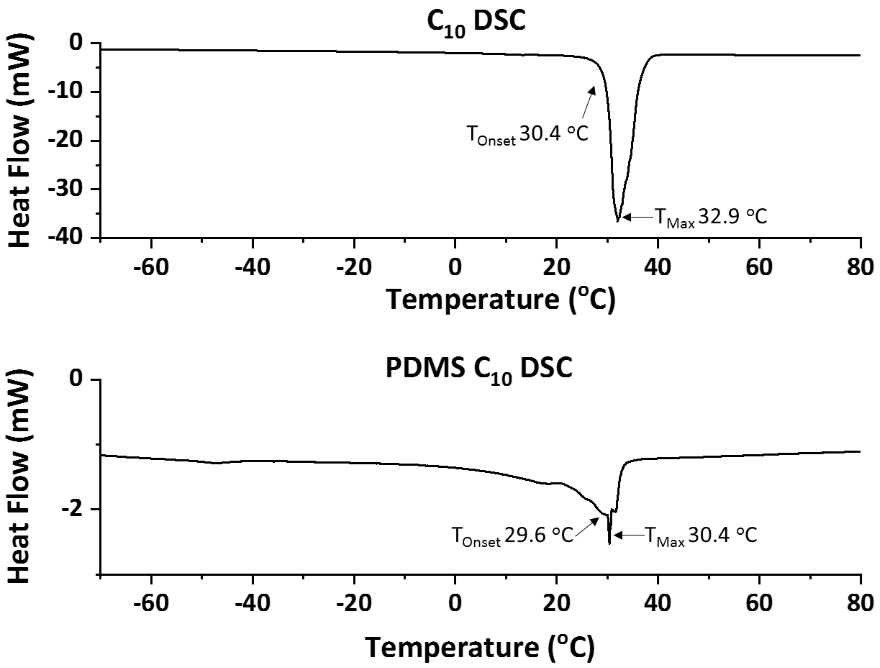

Figure SI12. Differential scanning calorimetry (DSC) profiles for decanoic acid (C_{10}) and PDMSC_{10} samples showing the endothermic peak of the fatty acid melting process. .(Exo – up)

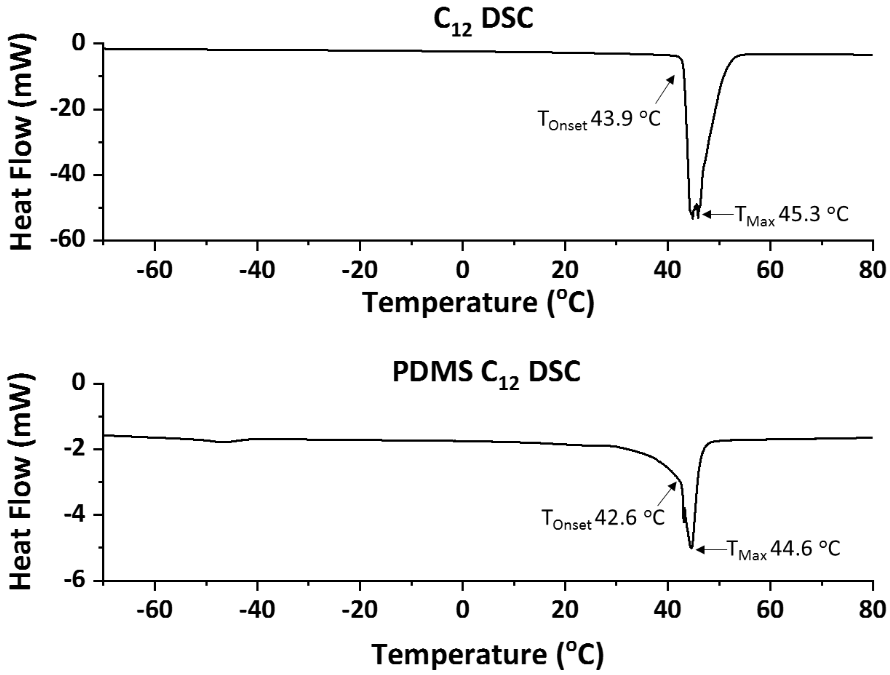

Figure SI13. Differential scanning calorimetry (DSC) profiles for Lauric acid (C_{12}) and PDMS C_{12} samples showing the endothermic peak of the fatty acid melting process. .(Exo – up)

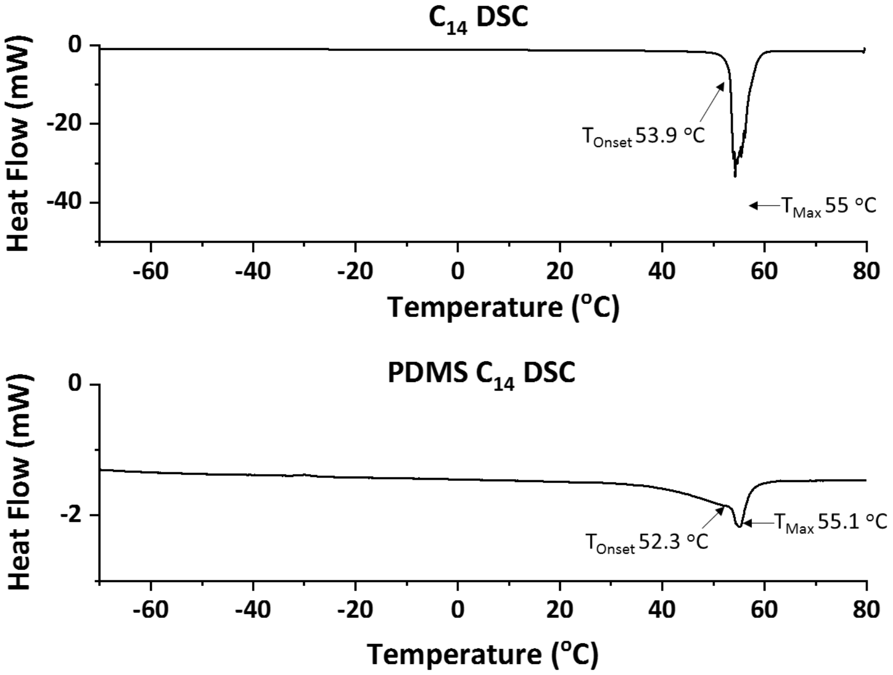

Figure SI14. Differential scanning calorimetry (DSC) profiles for myristic acid (C_{14}) and PDMS C_{14} samples showing the endothermic peak of the fatty acid melting process. .(Exo– up)

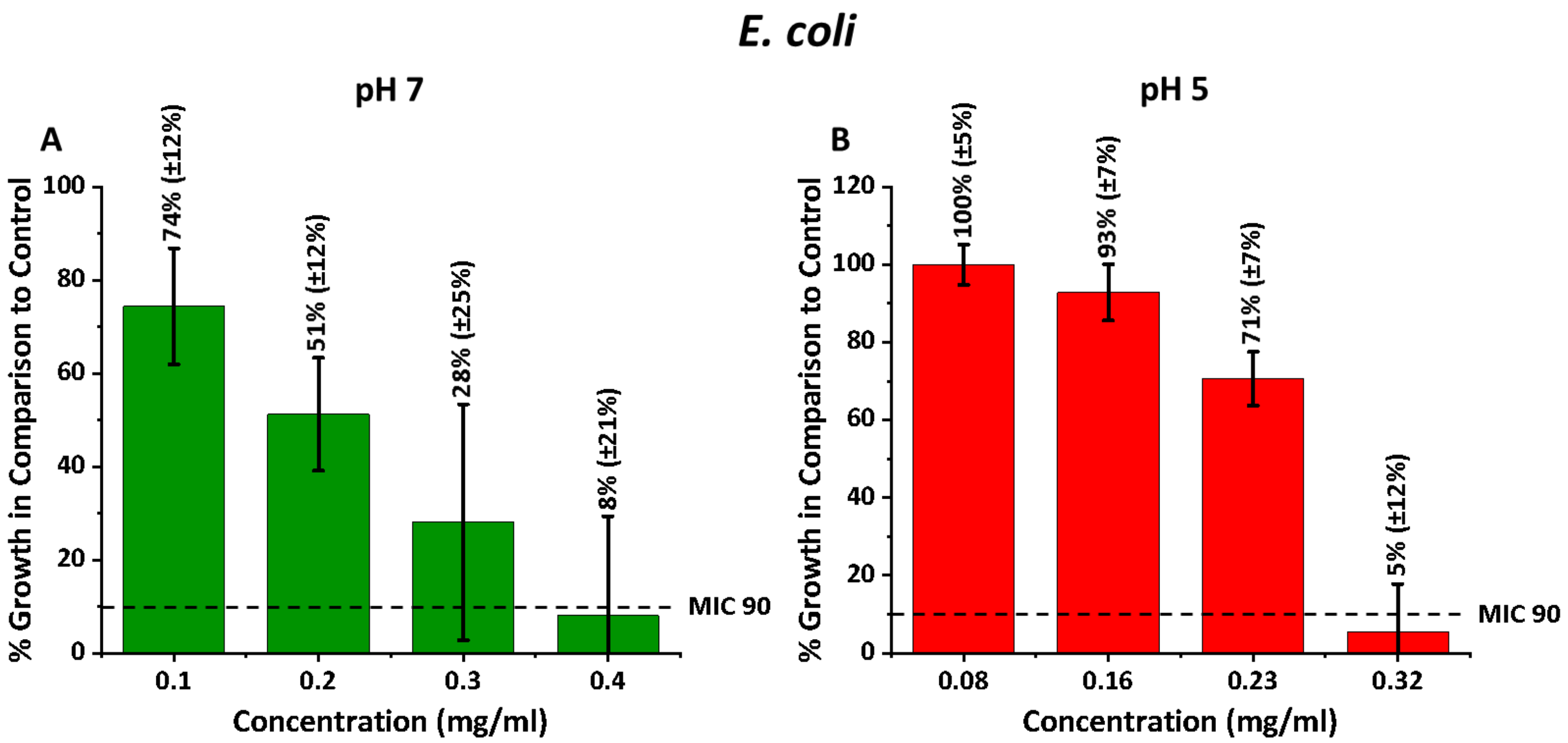

Figure SI15. Minimum inhibitory concentrations (MIC) for decanoic acid in LB media at different pH values. MIC90 was reached for 0.4 mg/ml and 0,32 mg/ml at pH=7 (A) and pH=5 (B) respectively.

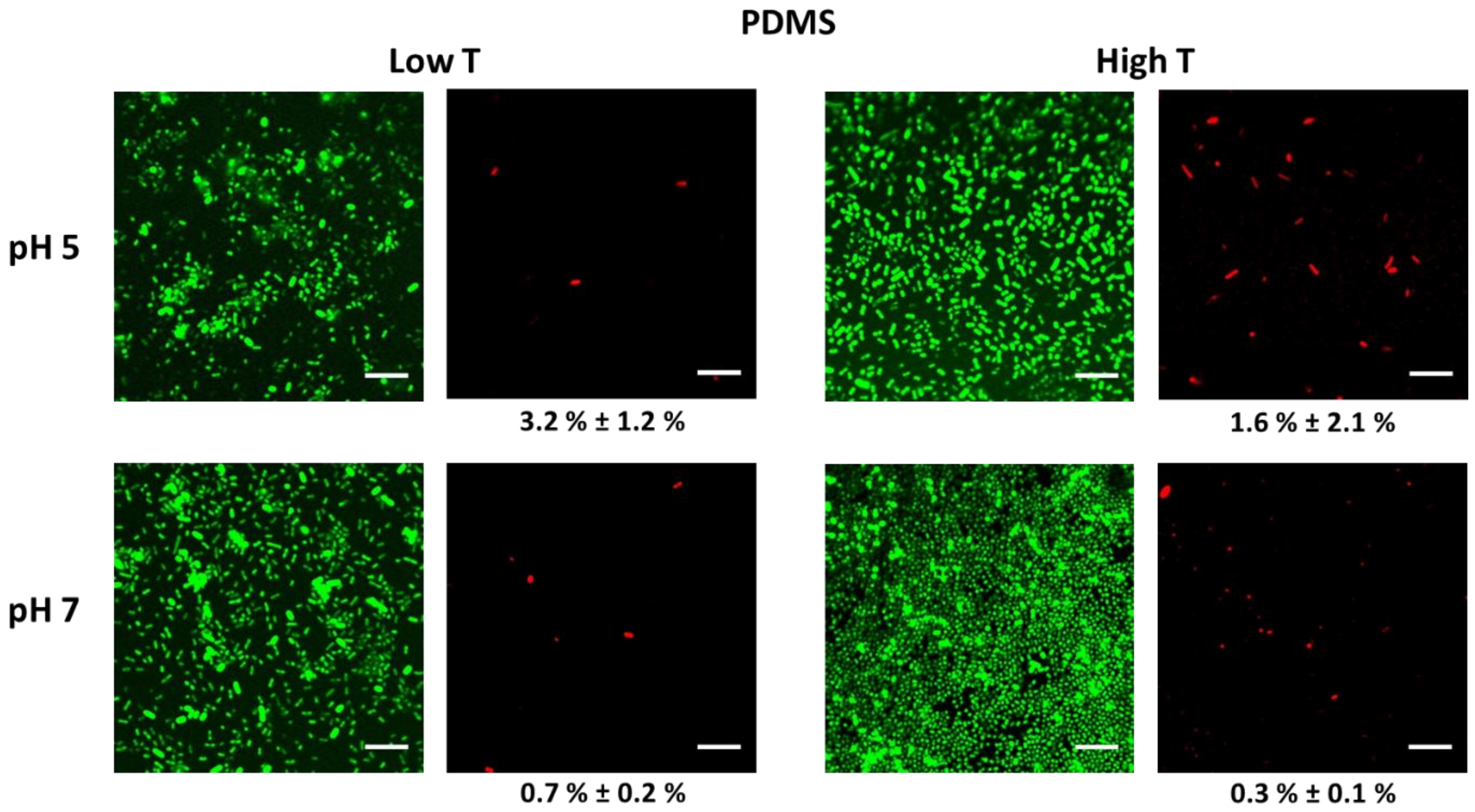

Figure SI16. Confocal Fluorescence Microscopy Data (Percentage Dead) of E. Coli 10798 on PDMS (Control). Bacteria Imaged Using Live/Dead Staining. (Green: All Bacteria), (Red: Dead Bacteria). (Scale Bar 10 μm).

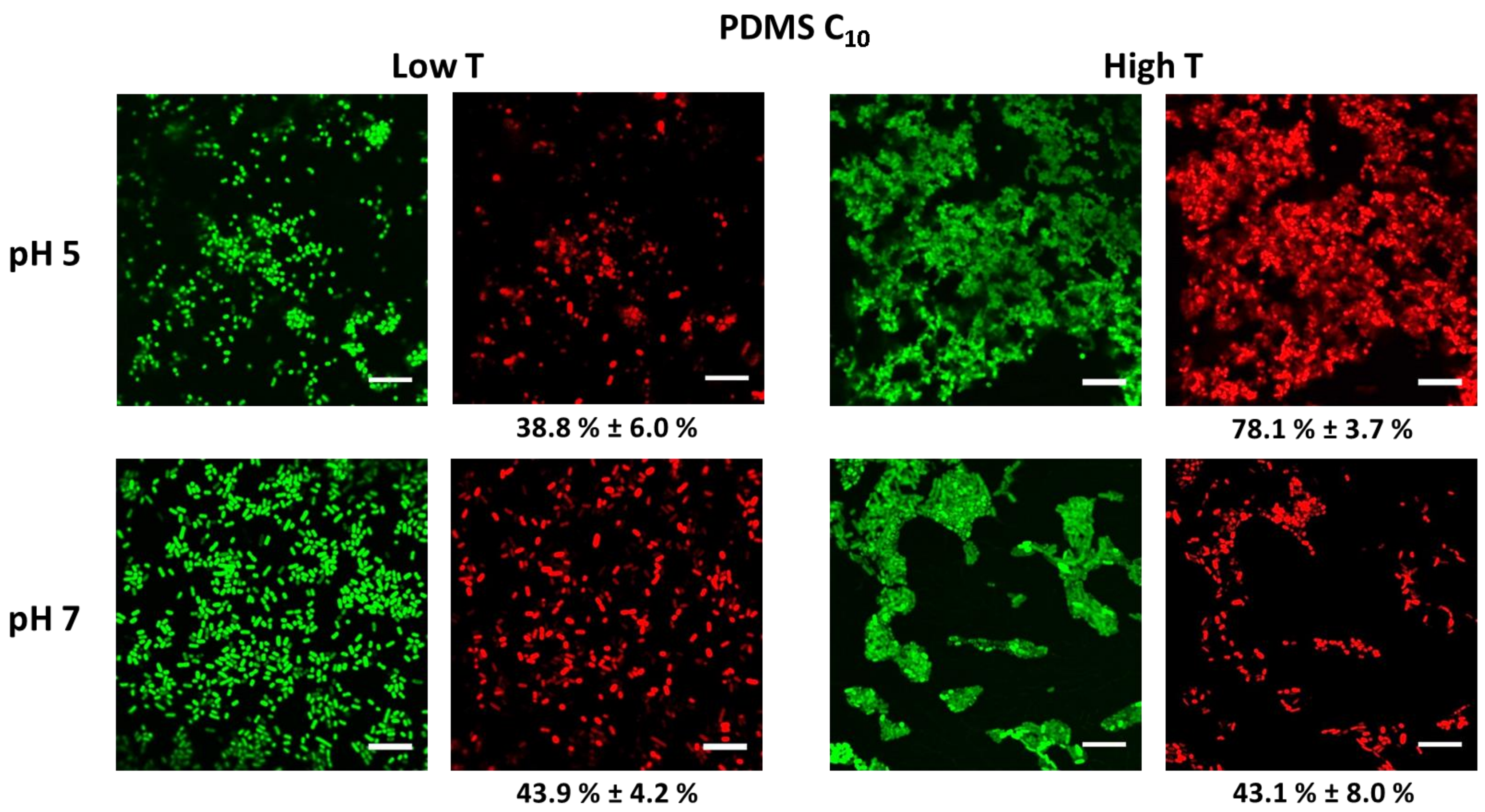

Figure SI17. Confocal Fluorescence Microscopy Data (Percentage Dead) of E. Coli 10798 on PDMS C_{10}. Bacteria Imaged Using Live/Dead Staining. (Green: All Bacteria), (Red: Dead Bacteria). (Scale Bar 10 μm).

Experimental Details

Chemicals: Sylgard 184 silicone elastomer kit (Dow Corning) was purchased from Univar Ltd. Hydrochloric acid (HCl, 37% w/v), glacial acetic acid (analytical grade), chloroform ($CHCl_3$, 99%) and ethanol (EtOH, absolute 99%) were purchased from Fisher Scientific. Sodium hydroxide (NaOH, 97%) and phosphate buffer saline (PBS) tablets were purchased from VWR. Lysogeny broth (LB) powder (Difco) was purchased from Scientific Laboratory Supplies (SLS). Live/ dead kit containing SYTO 9 and propidium iodide nucleic acid stains was purchased from ThermoFisher. All remaining chemicals listed were purchased from Sigma Aldrich: Decanoic acid (C_{10}, 98%), Lauric acid (C_{12}, 98%), Myristic acid (C_{14}, 98%), 4-(2-hydroxyethyl)-1-piperazineethanesulfonic acid (HEPES, 99.5%), sodium chloride (NaCl, 98%) and nutrient agar. All chemicals were used as received without further purification. Deionised water (Thermo Scientific Barnstead Smart2Pure), with a resistivity of 18 MΩ-cm was used throughout all experiments.

Fabrication of PDMS samples: PDMS was fabricated using Sylgard 184 silicone elastomer kit (Dow Corning). PDMS base and curing agent were mixed in a 10:1 ratio (base: curing (w/w)), followed by the addition of chloroform ($CHCl_3$) with a ratio of 2:1 (chloroform: base (v/w)). The mixture was then subject to vortex for 2 minutes to ensure homogeneity and pipetted (2.5 ml) into the wells of a non-stick baking tray. The tray was then loosely covered with aluminium foil and allowed to cure for 2 days in a fume hood at room temperature (RT) (approx. 25°C). The average weight of the PDMS material after curing was found to be 0.9055 g ± 0.088g.

Loading of PDMS samples with fatty acid: Decanoic (C_{10}), Lauric (C_{12}), and Myristic (C_{14}) acids were used to impregnate the PDMS matrix to form PDMS C_{10}, PDMS C_{12}, and PDMS C_{14} respectively. In a typical experiment, PDMS samples were weighed after curing and then impregnated with the fatty acid. The fatty acids were melted in individual glass jars at 37 °C (C_{10}), 50 °C (C_{12}), and 60 °C (C_{14}), forming a viscous liquid. PDMS samples were then swelled with the fatty acid by placing the cured PDMS samples in individual wells of a 6-well plate and covering them with 3 ml of melted fatty acid. The wells were covered with a lid, sealed with black Polyvinyl chloride (PVC) insulation tape and then placed in the oven at the required melting temperature (as above), overnight for a minimum of 16 hrs. After swelling the PDMS samples overnight, the samples in the well plate were placed immediately in the freezer, causing the fatty acids to solidify. To remove the samples from the solidified fatty acid in wells, the unincorporated fatty acid was broken with tweezers and the loaded PDMS sample removed. The fatty acid loaded PDMS materials were then washed quickly with ethanol and dried with compressed air to remove any additional unincorporated fatty acid, followed by weighing to determine the amount of fatty acid loaded in the material. We noted that during the various steps of the preparation process and analysis the PMDS loaded samples preserve their mechanical integrity and their ability to be manipulated did not change, suggesting that key mechanical properties of the material were not compromised.

Chemical characterisation of PDMS and fatty acid materials: Chemical characterisation of PDMS, fatty acids and the fatty acid loaded PDMS materials were analysed using Raman spectroscopy. Raman spectra were recorded with a Renishaw inVia confocal Raman microscope using an excitation wavelength of 532 nm and a 20x objective lens. The laser power and confocality used were kept constant for the individual comparable experiments. Raman

spectra were processed using WiRE 4.4 software (Renishaw), including cosmic ray removal and baseline correction.

Differential Scanning Calorimetry (DSC): The raw fatty acid and PDMS materials, along with the fatty acid loaded samples were weighed and then analysed using DSC. DSC data were acquired in air using a TA Instruments Q2000 differential scanning calorimeter, equilibrated to -70 °C and then run with a ramp rate of 5 °C min^{-1} to 80 °C. The DSC data was used to identify the average T_{Onset} (temperature onset of melting), T_{Max} (temperature of complete melting) and enthalpy of change (ΔH) of PDMS, raw fatty acids and fatty acid loaded PDMS materials. Pristine PDMS did not show DSC signals in the temperature range investigated (SI).

Determining the lateral XY distribution of fatty acid within the bulk (Z axis) of fatty acid loaded PDMS: The distribution of fatty acid within the bulk of the fatty acid loaded PDMS was investigated using Raman spectroscopy with a Renishaw inVia confocal Raman microscope with an excitation wavelength of 532 nm and x20 objective lens, using laser powers and confocality as previously mentioned. The laser power and confocality used were kept constant for the individual comparable experiments. Raman spectra were collected using a map image acquisition mode, at a depth of 500 µm from the sample surface, with a spectrum recorded every 75 μm^2, with a total of 100 acquisitions per sample across an area 1500 µm x 375 µm. All collected data were processed using WiRE 4.4 software (Renishaw) as described previous. A direct classical least squared (DCLS) component fit analysis,[9,10] with mean centre and scale to unit variance normalisation was used to determine the distribution of fatty acid in the PDMS.

Depth distribution of fatty acid loaded PDMS: The distribution of fatty acid across the depth of the material (surface to base) was spectroscopically examined using a Renishaw inVia confocal Raman microscope with an excitation wavelength of 532 nm and x20 objective lens, using laser powers and confocality as previously mentioned. Fatty acid loaded PDMS samples were first cut, placed on their side and the Raman spectra of the samples were collected using a map image acquisition mode, with a spectrum recorded every 5 μm starting from the top surface (Z_0) to the base of the material (Figure 3. 9. All collected data were processed using WiRE 4.4 software (Renishaw), with DCLS component analysis[9,10] as described for the lateral XY distribution.

Depth distribution of fatty acid loaded PDMS after re-heating under dry conditions: Fatty acid loaded samples were placed into 6 well plate, sealed with PVC tape and heated at their respective loading temperature to monitor the re-distribution of the incorporated fatty acid after 24 hrs. After 24 hrs the samples were cooled in the fridge for a minimum of 2 hours, allowing the fatty acid to re-solidify. The samples were analysed by Raman spectroscopy across the depth of the materials from the top surface (Z_0) to the base of the materials as described previous. All data was processed using WiRE 4.4 software (Renishaw), as previously described and the distribution of fatty acid was mapped using DCLS component analysis.[9,10]

Assessing the release of fatty acid from fatty acid loaded PDMS by gravimetric analysis: After loading of the PDMS with fatty acid, the samples were placed in wells of a 6 well plates with 3 ml of either pH 5 or pH 7 buffer, sealed with PVC tape and heated at a given temperature, to monitor the release of fatty acid after 24 hrs. The mass of the loaded PDMS samples used in these experiments were 0.9812 g ± 0.1168 g for PDMS C_{10}, 0.9666 g ± 0.1211 g for PDMS

C_{12} and 0.9343 g ± 0.0299 g for PDMS C_{14}. The release temperatures of the different samples were as follows: PDMS C_{10} samples at 5°C and 37°C; PDMS C_{12} samples at RT and 50°C; PDMS C_{14} samples at RT and 60°C. pH 5 buffer was made by using 1 M acetic acid in water and adjusting the pH with aliquots of 5 M NaOH. pH 7 buffer was made using 1 M HEPES in water and adjusting the pH using aliquots of 5 M NaOH. After 24 hrs the samples were cooled in the fridge for a minimum of 2 hours and then washed quickly with 6 ml of ethanol per sample, dried with compressed air and weighed. Gravimetric measurements were carried out using an AND HR-100AZ analytical balance.

Depth distribution of fatty acid loaded PDMS after release: The release of fatty acid from the fatty acid loaded PDMS samples and Raman spectroscopic analysis (including data processing) across the depth of the materials from the top surface (Z_0) to the base of the materials following the release were carried out as previously described. The distribution of fatty acid was mapped using DCLS component analysis.[9,10]

Static contact angle measurements: After loading of the PDMS with the individual fatty acids, the wettability of the samples was investigated using static contact angle measurements. pH 5 and pH 7 buffers as described above, were used for determining the contact angle of the samples. Contact angle measurements were measured using a First Ten Ångstroms (FTA) 1000 instrument with a Gilmont GS-1201 micrometre dispenser syringe via the sessile drop method. Contact angle images were collected with a frame rate of 60 frames S^{-1}. Contact angle data was then processed using FTA 32 drop shape analysis software (Version 2). The average contact angle of the samples was determined from 30 images per drop, for 3 drops on 3 different sample

replicates using a non-spherical fit. The contact angle of both buffers on pristine PDMS was measured as a control.

Preparation of bacterial cultures: *E. coli* (ATCC 10798) bacteria were transferred from frozen glycerol stock to a fresh nutrient agar plate and incubated overnight at 37°C. A minimum of three colonies were then taken from the agar plates and transferred to fresh LB (Lysogeny Broth) medium (Difco LB Broth Miller) and grown overnight in a shaking incubator (200 rpm, 37°C). The bacterial culture was subsequently refreshed by diluting with fresh LB media to starting concentration of 10^5 colony forming units (CFU) ml^{-1}.and incubated in a shaking incubator for 2-3 hrs until an optical density (OD) of 0.4 was achieved. This culture was then used for antimicrobial testing experiments.

Determining the minimum inhibitory concentration (MIC): Decanoic acid was chosen for antimicrobial testing due to its melting properties and PDMS release data. The solubility limit of decanoic acid in LB media was first determined before MIC testing could be conducted. Decanoic acid was weighed and added to LB media of both pH 5 and pH 7, which was then heated to 50°C and held at that temperature for 2-3 hrs to dissolve the decanoic acid. The natural pH of LB media was found to be pH 7. To achieve pH 5, the LB media was adjusted using 1 M HCl. The LB media with decanoic acid was then allowed to cool to room temperature and the solubility was determined by the formation of a precipitate. A solubility limit of 0.4 mg/ ml and 0.5 mg/ ml was achieved for decanoic acid in pH 5 and pH 7 LB media, respectively. The solutions were then filtered using 0.22 µm syringe filter. These solutions were then used for determining the MIC of *E. coli* (ATCC 10798) against decanoic acid, using LB media of both pH 7 and pH 5. Bacterial culture was prepared according to the above

conditions and then 100 μl of this was added to 4.9 ml of LB media of at either pH 5 or pH 7, with different concentrations of decanoic acid ranging from 0.1 mg/ml to 0.32 mg/ml and 0.1 mg/ml to 0.4 mg/ml for pH 5 and pH 7 respectively. pH-specific LB media, excluding decanoic acid was used as the control. The samples were then incubated for 24hrs at 37°C. After 24 hrs of incubation, the solutions were serially diluted and plated on fresh nutrient agar plates using the Miles and Misra method.[11,12] The plates were then incubated overnight at 37°C, and the CFUs were counted. This experiment was conducted in three biological replicates for each pH.

Viability of planktonic bacteria in contact with PDMS C_{10}: PDMS C_{10} samples were cut using a cork borer to a diameter of 7 mm. The cut samples were then placed in individual wells of a 6 well plate and covered with a PDMS frame to prevent the samples from floating. PDMS frames were fabricated in individual wells of 6 well plate using Sylgard 184 silicone elastomer kit (Dow Corning), at a ratio of 10:1 (base: curing agent). The mixture was stirred vigorously using a disposable spatula and then carefully poured into the individual wells of a 6 well plate, with 1g of mixture per well. The PDMS in the wells was then degassed under vacuum to remove trapped air bubbles and cured at 50°C overnight. The frame was formed by removing the cured PDMS from each well and cutting a 5 mm hole in the centre using a 5 mm cork borer. After carefully covering the PDMS C_{10} samples with the PDMS frames, the samples were sterilised by UV irradiation for 30 minutes on both sides. After sterilisation, 10 ml of pH-specific LB media, with *E. coli* (ATCC 10798) at a concentration of 10^5 CFU ml^{-1} was added to the individual wells of the 6 well plate with the PDMS C_{10} samples. Bacterial suspensions were prepared as described above (10^5 CFU ml^{-1}) with fresh pH specific LB media. Two sets of experiments were carried out simultaneously. One set of samples were incubated for 24 hrs at 37°C, at both pH 5 and pH 7. Another set of samples were incubated at 5°C, at both pH 5 and pH 7, with a starting concentration of 10^8 CFU ml^{-1}. After 24 hrs of incubation, the

planktonic suspension from each of the samples was collected, serially diluted and plated on fresh nutrient agar using the Miles and Misra method.[11,12] The agar plates were then incubated overnight at 37°C and the CFUs were counted. These experiments were conducted using three replicates for each condition: High T: High pH (37°C: pH 7), High T: Low pH (37°C: pH 5), Low T: High pH (5°C: pH 7) and Low T: Low pH (5°C: pH 5).

Viability of sessile bacteria in contact with PDMS C_{10}: The viability of sessile bacteria was probed using confocal fluorescence microscopy. PDMS C_{10} samples were incubated with *E. coli* (ATCC 10798) as described previous. After removing the planktonic suspension from the experiment discussed previous, with the frames still in place, the PDMS C_{10} samples were washed three times with 10 ml of sterile 0.85% (w/w) NaCl solution and then stained with live/dead BacLight bacterial viability kit (Molecular Probes, L7012). The staining process proceeded with the addition of 1 ml of sterile 0.85% (w/w) NaCl solution containing a mixture of 5 μM SYTO 9 (green-fluorescent nucleic acid stain for live cells), and 30 μM propidium iodide (red-fluorescent nucleic acid stain for dead cells). The samples were incubated in the dark for 15 minutes with the dyes and then immediately analysed in 0.85% (w/w) NaCl (as the immersion media) using a confocal upright Zeiss LSM 880 multiphoton microscope. The collected confocal fluorescence images were processed using Fiji software.[13]

Synchrotron X-ray powder diffraction: PDMS specimens encapsulated with decanoic, lauric, and myristic acids were measured at 10 keV (λ=1.23984 Å), in grazing incidence geometry (GIXRD), on the MCX beamline at the Elettra synchrotron light source in Trieste (Italy).[14] Samples were handled at low temperature (-20 °C) and quickly moved onto a Peltier-chamber sample-stage (see Figure SI22) kept at a constant temperature of -3 °C. After loading

the sample, the Peltier chamber was closed, and a protective argon atmosphere was applied at ~1 bar and kept throughout the entire duration of the experiment. Several glancing angles in the range θ=0.1-3° were investigated to study depth-dependence of the crystalline component. In the end, a glancing angle of 3° was chosen to get best signal from the encapsulated fatty acids (see Figures 5 and SI3-SI4). Synchrotron X-ray powder diffraction (SXRPD) patterns were collected at different temperatures, from -3 °C to 80 °C (or above the respective fatty-acids melting-points), to obtain information on the crystallinity within the polymeric matrix. A suitable temperature stabilization time was ensured before measuring. For comparison, SXRPD patterns of pristine samples (without PDMS) were collected on 0.7 mm glass capillaries using Debye-Scherrer geometry at the same energy and by spinning at 300 rpm.

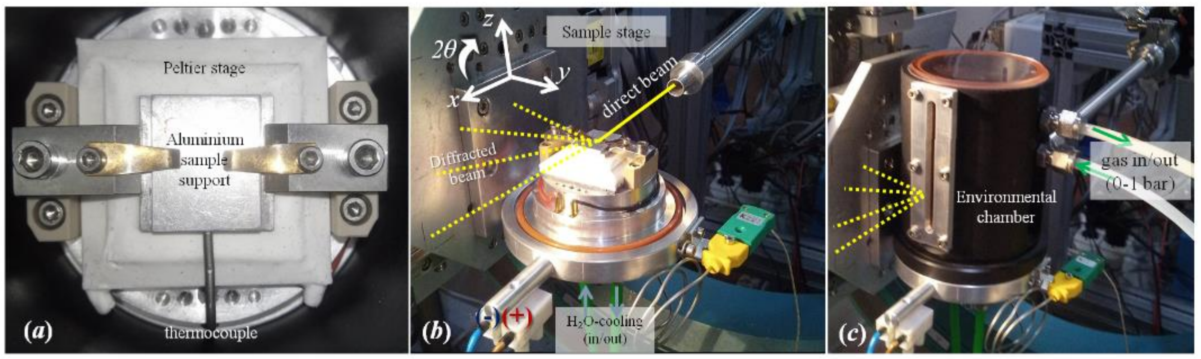

Figure SI18. Grazing-incidence XRPD setup on the MCX beamline. (a) Peltier sample support. (b) GIXRD experimental geometry. (c) Environmental chamber covering the Peltier stage for experiments under controlled atmosphere.

All three fatty acids in the PDMS matrix share the expected monoclinic symmetry with slightly different *b* and *c* lattice parameters and the longer perpendicular *a*-axis, the extension of which is highly dependent on the chain lengths.[15] The PDMS contribution to the overall GIXRD patterns is a merely amorphous background (Figures 5 and SI3-4).

References of Supporting Information

(1) Lide, D. R. Physical Constants of Organic Compounds. In *CRC Handbook of Chemistry and Physics*; Lide, D. R., Ed.; CRC Press, 2003; pp 3. 1-3.736. https://doi.org/10.1016/B978-1-907568-27-5.50012-3.

(2) Prisle, Nø. L.; Raatikainen, T.; Sorjamaa, R.; Svenningsson, B.; Laaksonen, A.; Bilde, M. Surfactant Partitioning in Cloud Droplet Activation: A Study of C8, C10, C12 and C14 Normal Fatty Acid Sodium Salts. *Tellus, Ser. B Chem. Phys. Meteorol.* **2008**, *60 B* (3), 416–431. https://doi.org/10.1111/j.1600-0889.2008.00352.x.

(3) Kanicky, J. R.; Shah, D. O. Effect of Degree, Type, and Position of Unsaturation on the PK a of Long-Chain Fatty Acids. *J. Colloid Interface Sci.* **2002**, *256*, 201–207. https://doi.org/10.1006/jcis.2001.8009.

(4) Gevantman, L. . Aqueous Solubility and Henry's Law Constants of Organic Compounds. In *CRC Handbook of Chemistry and Physics*; Lide, D. R., Ed.; CRC Press, 2003; pp 8. 92-8. 109. https://doi.org/10.1016/j.tetlet.2011.10.136.

(5) Eggenberger, D. N.; Broome, F. K.; Ralston, A. W.; Harwood, H. J. The Solubilities of the Normal Saturated Fatty Acids in Water. *J. Org. Chem.* **1949**, *14* (6), 1108–1110. https://doi.org/10.1021/jo01158a022.

(6) Alawi, S. M.; Akhter, M. S. Effect of N-Methyl Acetamide on the Critical Micelle Concentration of Aqueous Solutions of Some Surfactants. *J. Korean Chem. Soc.* **2011**, *55* (2), 163–168. https://doi.org/10.5012/jkcs.2011.55.2.163.

(7) Lee, S. Y.; Welbourn, R.; Clarke, S. M.; Skoda, M. W. A.; Clifton, L.; Zarbakhsh, A. Adsorption of Sodium Hexanoate on α-Alumina. *J. Colloid Interface Sci.* **2013**, *407*, 348–353. https://doi.org/10.1016/j.jcis.2013.06.019.

(8) Saggu, M.; Liu, J.; Patel, A. Identification of Subvisible Particles in Biopharmaceutical Formulations Using Raman Spectroscopy Provides Insight into Polysorbate 20 Degradation Pathway. *Pharm. Res.* **2015**, *32* (9), 2877–2888. https://doi.org/10.1007/s11095-015-1670-x.

(9) Zhou, Y.; Cao, H. An Augmented Classical Least Squares Method for Quantitative Raman Spectral Analysis against Component Information Loss. *Sci. World J.* **2013**, *2013* (Article 306937), 1–6. https://doi.org/10.1155/2013/306937.

(10) Galeano Díaz, T.; Guiberteau, A.; Ortíz Burguillos, J. M.; Salinas, F. Comparison of Chemometric Methods: Derivative Ratio Spectra and Multivariate Methods (CLS, PCR and PLS) for the Resolution of Ternary Mixtures of the Pesticides Carbofuran Carbaryl and Phenamifos after Their Extraction into Chloroform. *Analyst* **1997**, *122* (6), 513–517. https://doi.org/10.1039/a607955e.

(11) Miles, B. Y. A. A.; Misra, S. S. The Estimation of the Bactericidal Power of the Blood. *J. Hyg. (Lond).* **1931**, *38* (6), 732–749. https://doi.org/10.1017/S002217240001158X.

(12) Barbieri, L.; Sorzabal Bellido, I.; Beckett, A. J.; Prior, I. A.; Fothergill, J.; Diaz Fernandez, Y. A.; Raval, R. One-Step Preparation of Antimicrobial Silicone Materials Based on PDMS and Salicylic Acid: Insights from Spatially and Temporally Resolved Techniques. *npj Biofilms Microbiomes 2021 71* **2021**, *7* (1), 1–9. https://doi.org/10.1038/s41522-021-00223-6.

(13) Schneider, C. A.; Rasband, W. S.; Eliceiri, K. W. NIH Image to ImageJ: 25 Years of Image Analysis. *Nat. Methods 2012 97* **2012**, *9* (7), 671–675. https://doi.org/10.1038/nmeth.2089.

(14) Rebuffi, L.; Plaisier, J. R.; Abdellatief, M.; Lausi, A.; Scardi, A. P. MCX: A Synchrotron Radiation Beamline for X-Ray Diffraction Line Profile Analysis. *Zeitschrift für Anorg. und Allg. Chemie* **2014**, *640* (15), 3100–3106. https://doi.org/10.1002/ZAAC.201400163.

(15) Bond, A. D. On the Crystal Structures and Melting Point Alternation of the N-Alkyl Carboxylic Acids. *New J. Chem.* **2004**, *28* (1), 104–114. https://doi.org/10.1039/B307208H.

(16) Zalewski, K.; Chyłek, Z.; Trzciński, W.A.; Polymers **2021**, 13(7), 1080. doi: 10.3390/polym13071080
